# Characterization of constitutive CTCF/cohesin loci: a possible role in establishing topological domains in mammalian genomes

**DOI:** 10.1186/1471-2164-14-553

**Published:** 2013-08-14

**Authors:** Yuanyuan Li, Weichun Huang, Liang Niu, David M Umbach, Shay Covo, Leping Li

**Affiliations:** 1 Biostatistics Branch, National Institute of Environmental Health Sciences, Research Triangle Park, Durham, NC 27709, USA; 2 Department of Plant Pathology and Microbiology, Robert H. Smith Faculty of Agriculture, Food and Environment, Hebrew University, Rehovot, Israel

**Keywords:** CTCF, Cohesin, Constitutive binding site, Chromatin interaction, Topological domain

## Abstract

**Background:**

Recent studies suggested that human/mammalian genomes are divided into large, discrete domains that are units of chromosome organization. CTCF, a CCCTC binding factor, has a diverse role in genome regulation including transcriptional regulation, chromosome-boundary insulation, DNA replication, and chromatin packaging. It remains unclear whether a subset of CTCF binding sites plays a functional role in establishing/maintaining chromatin topological domains.

**Results:**

We systematically analysed the genomic, transcriptomic and epigenetic profiles of the CTCF binding sites in 56 human cell lines from ENCODE. We identified ~24,000 CTCF sites (referred to as constitutive sites) that were bound in more than 90% of the cell lines. Our analysis revealed: 1) constitutive CTCF loci were located in constitutive open chromatin and often co-localized with constitutive cohesin loci; 2) most constitutive CTCF loci were distant from transcription start sites and lacked CpG islands but were enriched with the full-spectrum CTCF motifs: a recently reported 33/34-mer and two other potentially novel (22/26-mer); 3) more importantly, most constitutive CTCF loci were present in CTCF-mediated chromatin interactions detected by ChIA-PET and these pair-wise interactions occurred predominantly within, but not between, topological domains identified by Hi-C.

**Conclusions:**

Our results suggest that the constitutive CTCF sites may play a role in organizing/maintaining the recently identified topological domains that are common across most human cells.

## Background

The CCCTC-binding factor (CTCF) is a C2H2-zinc finger protein with eleven zinc fingers that display close to 100% similarity between mouse, chicken, and human [1]. CTCF has a versatile role in genome regulation including transcriptional regulation, e.g., *c-Myc*[2,3], X chromosome inactivation [4], allele-specific silencing at imprinted loci such as *Igf2*/*H19*[5-8], and regulation of expression of lineage-specific gene clusters such as the β-globin locus [9] and the MHC class II locus [10]. Recently, CTCF has been implicated in splicing through its action on local RNA polymerase II pausing [11], trinucleotide repeat instability [12,13], DNA replication [14,15], and nucleosome positioning [16,17]. Because of these diverse functional roles in genome regulation, CTCF has been dubbed the “Master Weaver” of the genome [18].

CTCF sometimes co-localizes with cohesin [19,20]. Cohesin, a multi-subunit complex, consists of a heterodimer of SMC (structure maintenance of chromosomes) proteins, SMC1 (structural maintenance of chromosomes 1) and SMC3 (structural maintenance of chromosomes 3), with Rad21 [RAD21 homolog (*S*. *pombe*) also known as Scc1] and STAG (also known as Scc3). Cohesin was initially identified for its role in sister chromatid cohesion [14,21,22] but has been implicated in regulation of gene expression [19,20,23-27] and DNA replication [28,29]. Schmidt et al. have shown that cohesin can also bind to thousands of sites independent of CTCF [30].

The first genome-wide study of the CTCF binding in human cell lines identified ~14,000 CTCF binding sites [17]. Most of these sites were located far from the annotated transcriptional start sites (TSS). A subsequent analysis of CTCF binding in three human cell lines showed that they had around 40-60% of the CTCF binding sites in common [31]. Recently, Chen et al. [32] identified ~28,000 constitutive CTCF sites in 19 human cell types and showed that a large proportion of the variable CTCF binding between different cell types is linked to differential DNA methylation. A study of the evolution of CTCF binding in six representative mammals identified thousands of highly conserved, robust and tissue-independent CTCF binding sites [33]. Those studies suggest that, unlike many other transcription factors/proteins, a substantial portion of CTCF sites may be bound across multiple cell lines, *i.e.*, bound constitutively.

The canonical CTCF binding motif is 16 to 20 base pairs (bp) long [17]. Earlier studies suggested CTCF may be capable of binding to sequences of as long as 40–60 bp [2,3]. Footprinting of CTCF binding to the amyloid precursor protein (APP) promoter confirmed that CTCF can bind to a 40-bp fragment [34]. Recently, Schmidt et al. [33] identified a 33/34-mer full-spectrum CTCF motif in a subset of evolutionarily conserved CTCF binding loci.

Given its diverse functional roles in genome regulation, different “classes” of CTCF binding sites might exist where each class has a unique co-factor (or a combination of co-factors) and/or has different binding specificity (*e.g.*, canonical vs. full-spectrum). In this computational study, we examine the functional relevance of a unique “class” of CTCF binding sites – those that were constitutively bound across multiple human cell lines and co-localized with constitutively bound cohesin loci. We operationally refer to a binding site that was bound by a protein in 90% or more of available cell lines as a “constitutive” site.

Genome-wide CTCF-mediated chromatin interactions have been mapped using Chromatin Interaction Analysis Paired-End Tags (ChIA-PET) in mouse ES cells [24] and in K562 and MCF7 cell lines (data available at the ENCODE portal on UCSC genome browser). ChIA-PET identifies specific long-range chromatin interactions where widely separated genomic regions are brought to spatial proximity mediated by a protein detected by ChIP [35].

Recently, Guelen et al. [36] identified ~1300 sharply defined large domains defined by interactions with nuclear lamina components. Similarly, Dixon et al. [23] identified two to three thousand topological domains in multiple cell and tissue types by Hi-C [37]. Both studies suggested that human/mammalian genomes are partitioned into large, discrete domains that are units of chromosome organization. Dixon et al. [23] and Meuleman et al. [38] further proposed that these topological domains are common across different cell types and highly conserved across species. Both studies found that boundaries are enriched with CTCF sites. Those studies reinforce the notion that CTCF plays an important role in genome organization.

Using ChIP-seq (chromatin immunoprecipitation with sequencing) datasets from ENCODE [39] (http://genome.ucsc.edu/ENCODE/), we identified ~24,000 constitutive CTCF binding sites, ~12,000 of which further co-localized with constitutive cohesin loci. Our computational analysis further revealed that the CTCF-mediated chromatin interaction regions detected by ChIA-PET in both K562 and MCF7 cell lines were enriched with sites where constitutive CTCF and constitutive cohesin co-localized [24]. Furthermore, we found that these CTCF-mediated chromatin interactions were predominantly within topological domains rather than between them. Those results suggest that sites with constitutive CTCF plus constitutive cohesin may be involved in establishing/maintaining global chromatin structure that is common across cell lines [23,24].

## Results

### Definition

**Figure 1:**
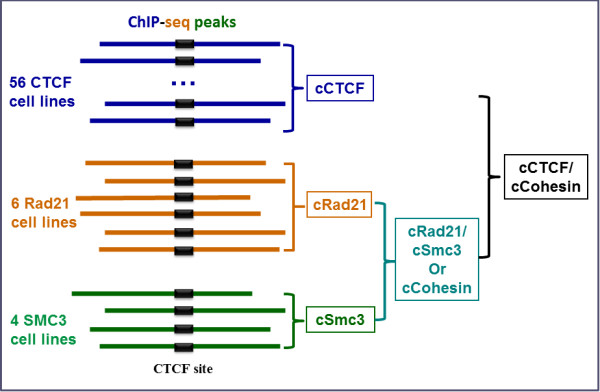
**A cartoon illustrating the various constitutive binding sites.** The blue, orange, and green horizontal bars represent the 200 bp ChIP-seq peaks for CTCF, Rad21, and Smc3 proteins, respectively. There were 56, 6, and 4 human cell lines for which CTCF, Rad21, and Smc3 ChIP-seq data were available. We first predicted the CTCF binding site in the peaks, represented by the black rectangles (see Method). We define as a “constitutive” CTCF site any CTCF binding site found in more than 90% of the cell lines. We use ‘c’ in front of a protein to indicate constitutive, ‘/’ between proteins to indicate co-localization/overlap. A cRad21 site that overlaps with a cSmc3 site is referred to as a cRad21/cSmc3 or cCohesin site, similarly for cCTCF/cCohesin sites.

ENCODE [39] makes available ChIP-seq data for CTCF as well as other proteins, with multiple cell lines available for some proteins. These data allowed us to identify CTCF binding sites within CTCF peaks and within ChIP-seq peaks for other proteins. We refer to a genomic site that is bound by a protein in more than 90% of cell lines as a “constitutive” site (Figure 1). (Our choice of 90% was arbitrary but designed to balance stringency with possible false negatives). We used those CTCF binding sites as surrogates both to identify constitutive CTCF loci and to identify, for *other proteins*, their constitutive loci that overlapped with constitutive CTCF loci (Figure 1).

Because all analyses involved CTCF binding sites in terms of constitutive sites, we simply refer to constitutive CTCF sites in CTCF peaks as constitutive CTCF sites, with CTCF here being the protein, not the motif. Throughout the manuscript, we used ‘c’ in front a protein to denote constitutive and ‘/’ between proteins to denote co-localization or overlap. For instance, a cCTCF/cRad21 site is a constitutive CTCF site within a CTCF peak that also lies within a constitutive Rad21 peak. We also use ‘site’ to indicate a motif site, as distinct from a ‘peak’ from ChIP-seq. We also refer to a broader region near a binding site as a locus.

### Constitutive CTCF sites

**Figure 2:**
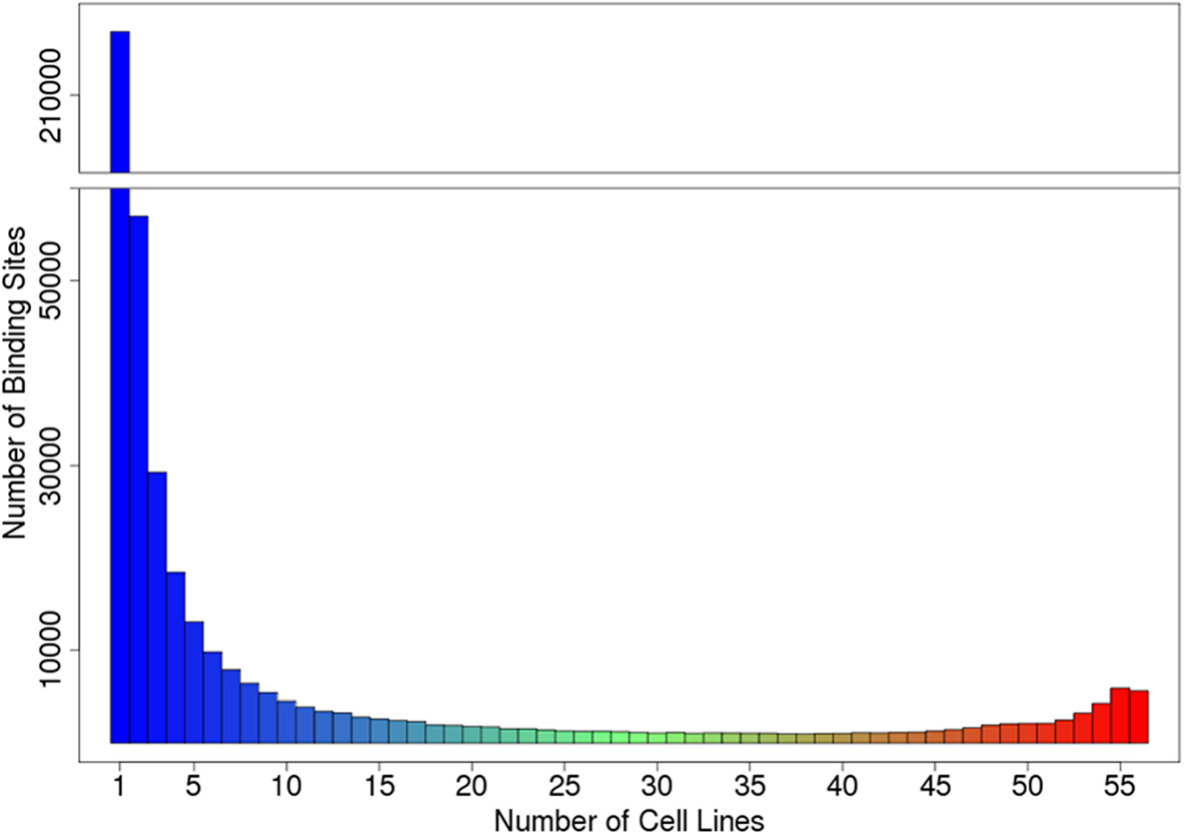
**Distribution CTCF binding sites according to the number of cell lines in which binding at the site was present.** Those at the right side of the plot are considered constitutive CTCF sites.

We identified 458,251 unique CTCF binding sites in the 112 human CTCF ChIP-seq datasets representing 56 cell lines (Figure 2). Over half of these sites (241,300 or 53%) were identified only in a single cell line, suggesting that those CTCF sites may be cell-line specific. Recently, Wang et al. [40] found that differential DNA methylation of the CTCF binding sites may count for a considerable portion of cell-selective regulation of CTCF binding. A substantial number (23,709) of CTCF sites were identified in more than 90% (≥ 51 of 56) of the cell lines, suggesting that those CTCF sites may be involved in some fundamental biological process common to most or all cell lines. One might consider the CTCF sites bound in all cell lines as the constitutive sites (5,611 in our data). ChIP-seq data is imperfect, however, so we regarded that criterion as too stringent – especially for questions of co-localization. Our goal was to probe the functional relevance of certain classes of CTCF sites, not to identify individual sites.

### Constitutive CTCF/cohesin sites

To characterize co-localization with cohesin, we predicted the CTCF binding sites in Rad21 peaks from 15 ChIP-seq datasets in six cell lines and in Smc3 peaks from four ENCODE ChIP-seq datasets in four cell lines (Gm12878, Helas3, Hepg2, and K562 cell lines). We identified 22,055 constitutive CTCF sites in Rad21 ChIP-seq peaks and 16,704 constitutive CTCF sites in Smc3 peaks (Table 1). Ninety per cent of the cSmc3 sites overlapped with the cRad21 sites. The majority of cRad21 sites (76%) and of cSmc3 sites (79%) overlapped with cCTCF sites (Table 1). We refer to the constitutive CTCF sites in CTCF peaks that overlap with the constitutive CTCF sites in both Rad21 and Smc3 peaks as the cCTCF/cCohesin sites (intersection of the three) (Table 1). The association of CTCF with cohesin is known [19,20], however, we refined that result by showing that this association was strongest when both are constitutive (Additional file 1, Additional file 2: Table S1-S3 and Additional file 3: Figures S1 and S2).

**Table 1:** Total CTCF binding sites in various classes of ChIP-seq loci

**Class name**	**Number of cell lines**		**Total binding sites**
	**Required**	**Available**	
Any CTCF	Any	56	458,251
CTCF in one cell line	1	56	241,300
cCTCF^1^	≥51	56	23,709
cRad21^1^	6	6	22,055
cSmc3^1^	4	4	16,704
cCTCF/cRad21	≥51/6	56/6	16,689
cCTCF/cSmc3	≥51/4	56/4	13,261
cCTCF/cCohesin	≥51/6/4	56/6/4	12,014

^1^To be declared constitutive, a site had to be identified in ≥90% of available cell lines.

Among the 23,709 cCTCF sites, 12,014 overlapped with cCohesin (cRad21/cSmc3) sites. Only 925 of the remaining 11,065 cCTCF sites did not overlap with any Rad21/Smc3 (cohesin) sites in the four cell lines with Smc3 ChIP-seq data available. We refer to these 925 sites as the cCTCF without any cohesin sites or cCTCF-non-cohesin sites and contrast their properties with those of cCTCF/cCohesin sites. Those cCTCF sites that overlap with cohesin loci in one, two or three of the four cell lines are not classified as either cCTCF/cCohesin or cCTCF-non-cohesin. (Additional file 4 list all sites that were constitutive by our criterion).

### cCTCF sites are more conserved than non-constitutive CTCF sites

**Figure 3:**
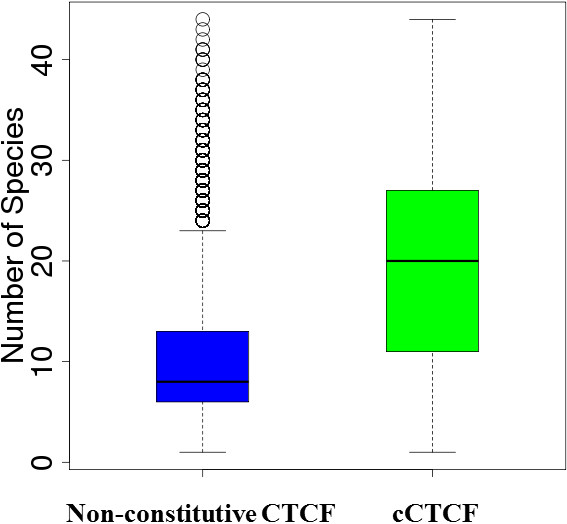
Boxplots of the number of species in which CTCF binding sites are found in the 46-way multiZ alignments for 23,709 cCTCF (green) and 23,709 randomly selected non-constitutive CTCF (blue) sites.

To see if cCTCF sites are more likely to be conserved than non-constitutive CTCF sites, we extracted the multiZ alignments of both the 23,709 human cCTCF and an equal number of randomly selected non-constitutive human CTCF sites and scanned for CTCF binding sites (see Methods). As expected, the cCTCF sites are twice more likely to be conserved among species than the con-constitutive CTCF sites (Figure 3). On average, cCTCF sites could be found in 20 species compared to 10 for non-constitutive CTCF sites.

### Most cCTCF/cCohesin sites are distant from promoters and lack CpG islands

**Figure 4:**
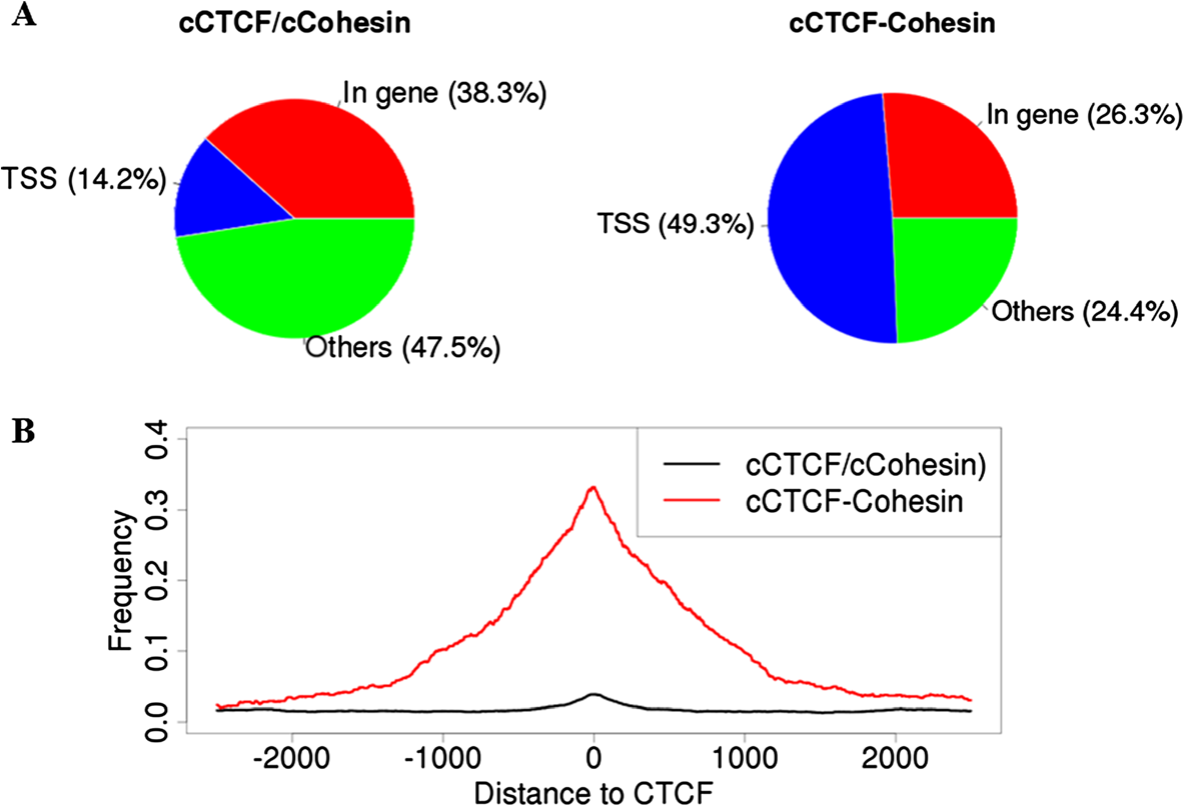
**Genomic distributions of the 12,014 cCTCF/cCohesin and the 925 cCTCF-cCohesin sites. (A)** Pie charts. If a binding site is within ±5 kb from the transcription start site (TSS) of a reference gene (GRCh37), it was annotated as ‘TSS’. Otherwise, if it is located between the TSS and the transcription end site of a gene, it was annotated as ‘in gene’. All others were annotated as “others”. **(B)** The density of CpG islands. For each CTCF binding site, we computed the cumulative density of the CpG islands within ±2.5 kb centered at the site.

Most cCTCF/cCohesin sites were located in intergenic or intronic regions, away from transcription start sites (TSS) and without CpG islands (Figure 4). The lack of CpG islands near the cCTCF/cCohesin sites is consistent with the finding that a considerable portion of the cell-type variable CTCF binding sites are subject to differential DNA methylation [32]. DNA methylation in CpG islands near CTCF binding sites affects CTCF binding and, subsequently, transcription regulation of the nearby enhancer elements [5,41]. Together, those results suggest that the role of the cCTCF/cCohesin sites may be structural rather than transcriptional. In contrast, the cCTCF-non-cohesin sites tend to be located near TSS and in CpG islands (Figure 4).

### cCTCF/cCohesin loci are associated with constitutive open chromatin

We examined other proteins for possible association with cCTCF loci. In each cell line separately, we counted how often a protein had the center of its ChIP-seq peak within ± 200 bp from CTCF sites in three classes: the 12,014 cCTCF/cCohesin sites, the 925 cCTCF-non-cohesin sites, and all CTCF sites bound in the given cell line but excluding the cCTCF sites. We used all ENCODE TFBS datasets (encodeHaib, encodeSydh, encodeUTA, and encodeUW), histone modification datasets (encodeHistoneBroad, encodeHistoneUW, encodeHistoneUTA), and open chromatin datasets (encodeDukeDNase, encodeUWDNase, and encodeUNCFaire). The total number of histone marks analysed was 11 (Additional file 1). CTCF, Rad21, and Smc3were not included in the analysis. In total, we included 1011 ChIP-seq datasets representing 68 factors in this analysis and combined results from all cell lines for the same factor/feature.

**Figure 5:**
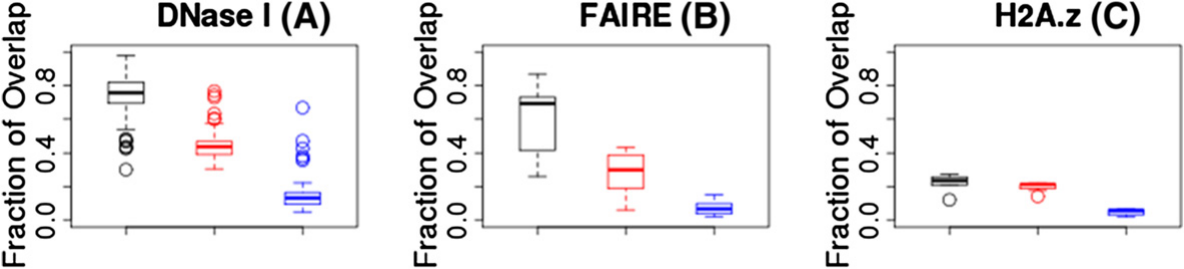
**Box plots of the fraction of overlap within each cell line between classes of CTCF sites and open chromatin (as assessed by either DNase I or by FAIRE) or H2A.z.** The classes of CTCF sites are: cCTCF/cCohesin sites (black), cCTCF-non-cohesin sites (red), or all CTCF sites bound in each cell line excluding cCTCF sites (blue). Each point in a box plot represents a cell line.

On average, 74% of the cCTCF/cCohesin sites overlapped with DNase I [42,43] peaks compared to only 44% of the cCTCF-non-cohesin sites or 15% of all CTCF sites bound in a given cell line excluding cCTCF sites (Figure 5A); the same pattern held with FAIRE [44,45] peaks (Figure 5B). These results together indicate that cCTCF/cCohesin sites are highly associated with open chromatin. H2A.z was also more likely to be associated with the cCTCF compared to all CTCF sites bound in a given cell line excluding cCTCF sites (Figure 5C). Several factors, especially those that tend to be associated with actively transcribed genes or located at the proxy promoter such as H3k4me3 were more likely to be associated with the cCTCF-non-cohesin sites (Additional file 3: Figure S3).

### cCTCF/cCohesin loci are enriched with the 33/34-mer and 20/26-mer motifs

The motif logo of the extended cCTCF/cCohesin sites (30 bp at each side) showed information flanking the 16-bp core CTCF motif (data not shown), indicative of the existence of additional motifs. To discover those motifs, we used the 12,014 cCTCF/cCohesin sequences. At each of the 30+30+16-5+1=72 positions, we counted the number of sequences in which each of the 1,024 possible *k*-mers (*k*=5) occurred as in [46]. The counts were then ranked and the top 50 *k-*mers at each side of the core CTCF motif were combined separately to create the composite motif for the side using the position-specific *k*-mer frequency as the weight.

**Figure 6:**
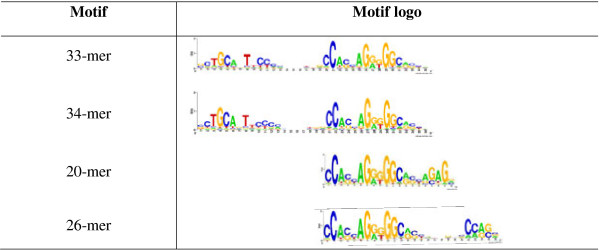
Motif logos of the 33/34-mer and 20/26-mer motifs.

This process identified a 33/34-mer full-spectrum CTCF motif (Figure 6) that was reported previously [30]. In addition, we identified two potentially novel motifs at the other side of the core CTCF motif (Figure 6). It is unclear if the pattern represents two separate motifs (denoted the 20-mer and the 26-mer) or one single motif (the 20+26-mer). The extra motif in the 20-mer resembled the GAGA-binding motif (GAGA) whereas the extra motif in the 26-mer resembled that for CAF1 (chromatin assembly factor) according to STAMP (http://www.benoslab.pitt.edu/stamp/). All of these full-spectrum motifs showed higher enrichment among cCTCF/cCohesin loci compared to either cCTCF-non-cohesin or all CTCF loci (Table 2). It is not clear if the additional motifs (20-/26-mer) at the right side of the canonical motif are bound by CTCF or by a co-factor. This question needs to be determined experimentally.

**Table 2:** Proportions of the 33/34-mer and the 20/26-mer motifs in various classes of CTCF loci

**Motif**	**cCTCF/cCohesin**	**cCTCF-non-cohesin**	**All CTCF sites**
33-mer	14.8%	3.8%	1.8%
34-mer	11.3%	3.8%	1.7%
20-mer	8.2%	3.7%	1.2%
26-mer	6.8%	2.1%	1.1%

Recently, additional CTCF motifs such as (C6D, C7D, C8D, and U5C7D) have been reported [47]. However, the proportions of those motifs in the context of the core motif are low ranging from 0.3% to 4% in CD43 cells. We estimated that the proportions of those motifs in both cCTCF and non-constitutive CTCF loci were ~0.5%.

### cCTCF is enriched in CTCF-mediated chromatin interactions

Several studies have implicated CTCF in mediating long-range chromatin interactions [18-20,23-26,48,49]. The genome-wide CTCF-mediated chromatin interactions in cell lines K562 and MCF7 have been mapped using Chromatin Interaction Analysis Paired-End Tags (ChIA-PET) from ENCODE/GIS-Ruan. In cell line K562, we identified 105,041 unique CTCF binding sites (combined from replicates) in CTCF ChIP-seq peaks, among which 23,577 were cCTCF sites. The proportion of ChIA-PET interaction sites among these cCTCF sites was significantly higher than the corresponding proportion among the non-constitutive CTCF binding sites (odds ratio=7.7, *p*-value≈0, one-sided Fisher exact test) (Table 3a). This enrichment was also seen for cell line MCF7 (odds ratio=8.5, *p*-value≈0, one-sided Fisher exact test) (Table 3b). The majority of the cCTCF sites (60% and 82%, in K562 and MCF7, respectively) overlapped with the chromatin interaction regions compared to only 16% and 35% of the non-constitutive CTCF sites. Similarly, we found that among all CTCF-mediated interactions, more interactions involved cCTCF than the non-constitutive CTCF in both K562 and MCF7 cell lines (Table 4), respectively, despite there being 2.5 and 1.9 times more non-constitutive CTCF sites than cCTCF sites in those cell lines. Together those results suggest that chromatin interactions may be mediated largely by cCTCF sites.

**Table 3:** Proportions of various CTCF binding sites that contained within the CTCF-mediated interaction regions from ChIA-PET

**Comparison**	**Total sites**	**Sites in interaction**		**Odds ratio**	***P*-value**
		**Number**	**Proportion**		
**(A) K562**					
cCTCF	23,577	14,178	0.60	7.7	~0
All CTCF excluding cCTCF	81,464	13,316	0.16		
cCTCF/cCohesin	12,014	8,714	0.73	2.9	~0
cCTCF excluding cCohesin	11,563	5,464	0.47		
**(B) MCF7**					
cCTCF	23,641	19,398	0.82	8.5	~0
All CTCF excluding cCTCF	67,752	23,701	0.35		
cCTCF/cCohesin	12,001	10,796	0.90	3.2	4×10^-227^
cCTCF excluding cCohesin	11,640	8,602	0.74		

**Table 4:** Proportions of CTCF-mediated interactions involving cCTCF and non-constitutive CTCF sites

**Category of interaction**		**Count (%)**
**CTCF type in region 1/2**	**CTCF type in region 2/1**	
**(A) K562**		
cCTCF	cCTCF	8,484 (33.5%)
cCTCF	Neither	3,107 (11.9%)
cCTCF	Any CTCF excluding cCTCF	8,991 (35.5%)
Any CTCF excluding cCTCF	Any CTCF excluding cCTCF	2,567 (10.1%)
Any CTCF excluding cCTCF	Neither	1,627 (6.4%)
Neither	Neither	617 (2.4%)
**(B) MCF7 replicate 1**		
cCTCF	cCTCF	13,176 (26.1%)
cCTCF	Neither	6,590 (13.1%)
cCTCF	Any CTCF excluding cCTCF	17,601 (34.9%)
Any CTCF excluding cCTCF	Any CTCF excluding cCTCF	6,738 (13.3%)
Any CTCF excluding cCTCF	Neither	4,265 (8.5%)
Neither	Neither	2,127 (4.2%)
**(C) MCF7 replicate 2**		
cCTCF	cCTCF	6,026 (29.9%)
cCTCF	Neither	2,332 (11.6%)
cCTCF	Any CTCF excluding cCTCF	6,693 (33.2%)
Any CTCF excluding cCTCF	Any CTCF excluding cCTCF	2,219 (11.0%)
Any CTCF excluding cCTCF	Neither	1,469 (7.3%)
Neither	Neither	1,401 (7.0%)

Because of the strong association between cCTCF and cCohesin in K562 and MCF7 cell lines, we found that the odds of ChIA-PET detected interactions were approximately 3 times greater among the cCTCF/cCohesin sites than among the cCTCF sites without cCohesin (*p*-value≈0) (Table 3).

### Interplay between topological domains and cCTCF sites

Two recent studies [23,36] suggested that human/mammalian genomes are divided into large, discrete domains that are units of chromosome organization. Dixon et al. [23] further proposed that the topological domains are common across different cell types and highly conserved across species. Those results, together with our ChIA-PET results, suggest that chromatin topological domains and CTCF-mediated chromatin interactions may be intrinsically linked.

**Figure 7:**
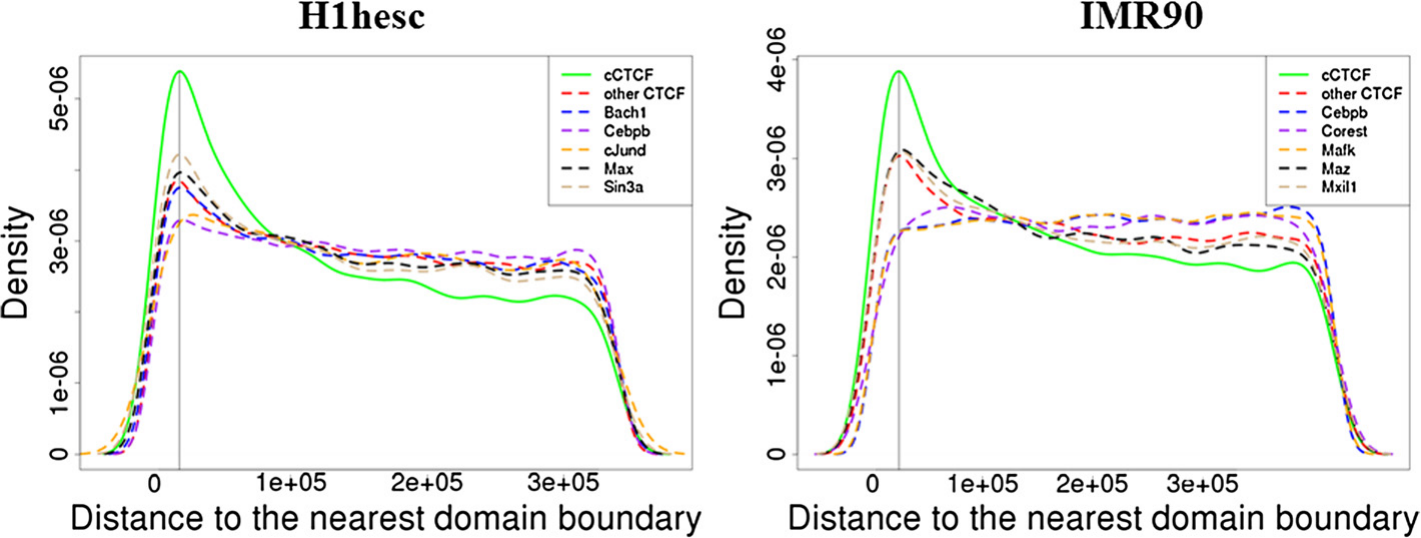
**Kernel density estimate of the distances (in bp) between CTCF sites (cCTCF in green and all CTCF sites excluding the cCTCF in red) and their nearest topological domain boundaries.** Since more than 90% of the CTCF sites are located inside topological domains, only CTCF sites located inside topological domains were included. We standardized all distances to the median length of all domains in each cell line (680 kb in H1hesc and 840 kb in IMR90). The distances from the nearest boundary at maximum density (vertical lines) are ~19 kb and 25 kb for H1hesc and IMR90 cell lines, respectively.

Indeed, we found that 87% of the CTCF-mediated interactions from ChIA-PET in either K562 or MCF7 cell line were within the topological domains of H1hesc cell line (mean and median domain lengths 852 kb and 680 kb) [23]. Importantly, among those CTCF-mediated intrachromosomal interactions that were in the topological domains, 77% to 81% had both regions of the pair in the same domain, suggesting that the CTCF-mediated chromatin interactions are predominantly within a domain. More than 90% of both cCTCF and non-constitutive CTCF sites were located inside the topological domains. However, the non-constitutive CTCF sites tended to be uniformly distributed within the domain whereas the cCTCF sites appear to be enriched near the boundaries of the domains (Figure 7). As a control, we selected five other ENCODE TFBS ChIP-seq datasets with the highest number of peaks (Bach1, Cebpb, cJund, Max, and Sin3a for K562 and Cebpb, Corest, Mafk, Maz and Mxil1 for Imr90) and computed the distances between the ChIP-seq peaks and the nearest topological domains. Similar to the non-constitutive CTCF sites, the distribution of the distances for the five proteins in each cell is more uniform than the corresponding distribution for cCTCF (Figure 7).

## Discussion

CTCF is a multi-functional protein that has been implicated in transcriptional regulation, insulation, DNA replication, X-chromosome inactivation, splicing chromatin packaging and many others [18]. CTCF binding sites are widespread in genomes from fly to humans [1,18]. Earlier, several genome-wide studies identified ~14,000 to ~27,000 CTCF binding sites in several human cell lines. Those studies also showed that 40-60% of the CTCF sites in the cell lines studied were invariant to cell types [17,31]. Many CTCF binding sites were also computationally identified [50] and found to be conserved [17,31,33,50,51]. However, it remained unclear how many CTCF binding sites are present in the human genome and what proportion of them is constitutively bound across most cell lines/tissues. A comprehensive CTCF binding site database containing more than 15 million sequences in 10 species has been recently updated to include long-range chromatin interaction data mediated by CTCF [52], thereby facilitating analyses like ours in non-human species.

Our analysis of 112 ENCODE CTCF ChIP-seq datasets representing 56 human cell lines suggests that there might be as many as 450,000 CTCF binding sites in the human genome. Nearly half were found in CTCF peaks in only one of the 56 cell lines. About a quarter million of the CTCF sites were found in CTCF peaks in more than one of the 56 cell lines. Moreover, ~24,000 CTCF binding sites were found in CTCF peaks in more than 90% (at least 51 of 56) cell lines, suggesting that those constitutive CTCF sites may be implicated in some fundamental biological process/function for most or all cell lines.

Of course, the exact numbers of cCTCF sites identified by our methods depend on thresholds used for making decisions. In our analysis, we trimmed/extended all peaks to 200 bp in length from the center. Using 300 bp instead increased by 1,640 the number of CTCF sites declared constitutive. Including these additional sites in our analysis of ChIA-PET interactions yielded results substantially the same as those in Table 4. In our analysis, a *p*-value cut-off of 0.0005 on the PWM score identified a CTCF binding site in 80-95% of the peaks. Adjusting the cut-off would certainly affect the number of CTCF sites identified and declared constitutive; but, like changing the peak length, changing this cut-off seems unlikely to influence our results about enrichment and our overall conclusions about the role of cCTCF sites.

Because many datasets used in our analysis were from cancer cell lines which often carry genetic and chromatin aberrations, we looked for evidence that cCTCF sites might diverge between cancer and normal cell lines. We identified 27,735, 28,662, and 27,774 cCTCF sites in recently deposited CTCF ChIP-seq from 23 cancer cell lines, 20 normal cell lines, and 19 cell lines with unknown karyotypes, respectively [40]. Not only did these three groups have similar numbers of cCTCF sites, they had 19,279 (80.5% to 83.2%) cCTCF sites in common, indicating that cell origins have little effect on the number or locations of cCTCF sites.

The nature of ChIP-seq experiments is to capture a snapshot of protein binding in time. Thus, the sites that we define as constitutive because they are bound in over 90% of cell lines are likely sites where a protein spends most time in the bound state -- perhaps an individual binding event of long duration or perhaps frequent bouts of binding/unbinding with the bound state predominating. Long-duration binding might be attributed to strong binding whereas frequent binding/unbinding would not be. Thus, the constitutive sites that we detect should not correspond exactly to sites with strong binding, though different binding motifs (canonical vs. full-spectrum) might be correlated with binding strength. On the other hand, one can imagine sites in the genome where a protein bound relatively briefly but the site is bound at some time in every tissue or cell line. Such a site would theoretically meet our definition of ‘constitutive’ but would go undetected by our analysis as ChIP-seq snapshots would be virtually impossible to capture short-term binding at the same site in multiple cell lines.

Strong binding may occur at constitutive sites, but it may not be the only explanation for their existence. We recently developed an alternative method for identifying constitutive sites using peak data only (without motif search) (manuscript in preparation). We identified constitutive sites for 22 factors with ChIP-seq data in more than six cell lines. We found that the proportions of constitutive sites vary between different factors from a few to many thousands. It is unlikely that factors that bind to the highest number of constitutive sites (e.g., CTCF and Rad21) are strong binders whereas those that bind to the fewest constitutive sites (e.g., JunD) are weaker binders. We also found that gene ontology analysis of the target genes of the constitutive Pol II sites are highly enriched with biological processes such as metabolism and cell cycle (data not shown). Together, those results strongly suggest that the constitutive sites are biologically meaningful.

Because of CTCF’s diverse roles in genome regulation, different “classes” of CTCF binding sites might exist to carry out different functional roles. Such classes might differ in their co-factors and/or binding strength and specificity (e.g., canonical vs. full-spectrum motifs). In this study, we focused on the class of CTCF binding sites that are constitutively bound and co-localized with the constitutive cohesin loci and compared it to a class of constitutive CTCF binding sites without cohesin. We examined the genomic features, transcriptional landscape and epigenetic environments of those sites to gain insights into their functional relevance. Our analysis not only included many more datasets but also was more comprehensive than the earlier analyses of CTCF binding sites [16,31,32,51,53].

We identified ~12,000 constitutive CTCF binding sites co-localized with constitutive cohesin loci. The majority of these cCTCF/cCohesin sites were located ≥ 5 kb from the TSS in introns or in intergenic regions that lacked CpG islands. Furthermore, the cCTCF/cCohesin loci were enriched in H3k4me1 mark with well-positioned nucleosomes (Additional file 1). A substantial number of the cCTCF sites overlapped with cohesin in one or more cell lines without meeting the criterion that the corresponding Rad21 and Smc3 peaks were in ≥ 90% of available cell lines. In contrast, few cCTCF sites did not co-localize with cohesin loci in any cell line.

Our analysis of the constitutive sites is limited by the number of cell lines studied; some factors have data from only a limited number of cell lines. As data from additional cell lines become available, some of the cCTCF/cCohesin sites will no longer be designated as constitutive. Although the cCTCF sites were found in at least 51 of the 56 cell lines, constitutive cohesin was defined via Rad21 and Smc3 peaks, which were identified in only 6 and 4 cell lines, respectively.

Numerous studies have shown that CTCF cooperates with cohesin to contribute to DNA loop formation to thereby regulate gene expression and chromatin interactions [18-20,23-26,48,49,54], DNA replication [14], RNA pol II pausing [11]. Our computational analysis revealed that the strength of association between CTCF and cohesin increases when both sites/loci were constitutive, similarly for CTCF and Znf143 (Additional file 1 and Additional file 2: Table S2), and for CTCF, cohesin, and Znf143 (Additional file 1 and Additional file 2: Table S3).

A footprinting study of CTCF binding to the promoter of the APP gene showed that the binding of the full-length CTCF protein generated a DNase I protected region covering 40 bp [34]. Subsequent motif analysis [33] in a set of evolutionarily conserved CTCF sites identified ~5,000 33/34-mer full-spectrum CTCF binding sites. We independently identified the same 33/34-mer motifs in the set of cCTCF/cCohesin loci. Furthermore, we also identified two potentially novel 20/26-mer CTCF motifs (Figure 6). Whether those full-spectrum motifs function in transcriptional regulation or in mediating chromatin-chromatin interactions, or both, remains unclear.

**Figure 8:**
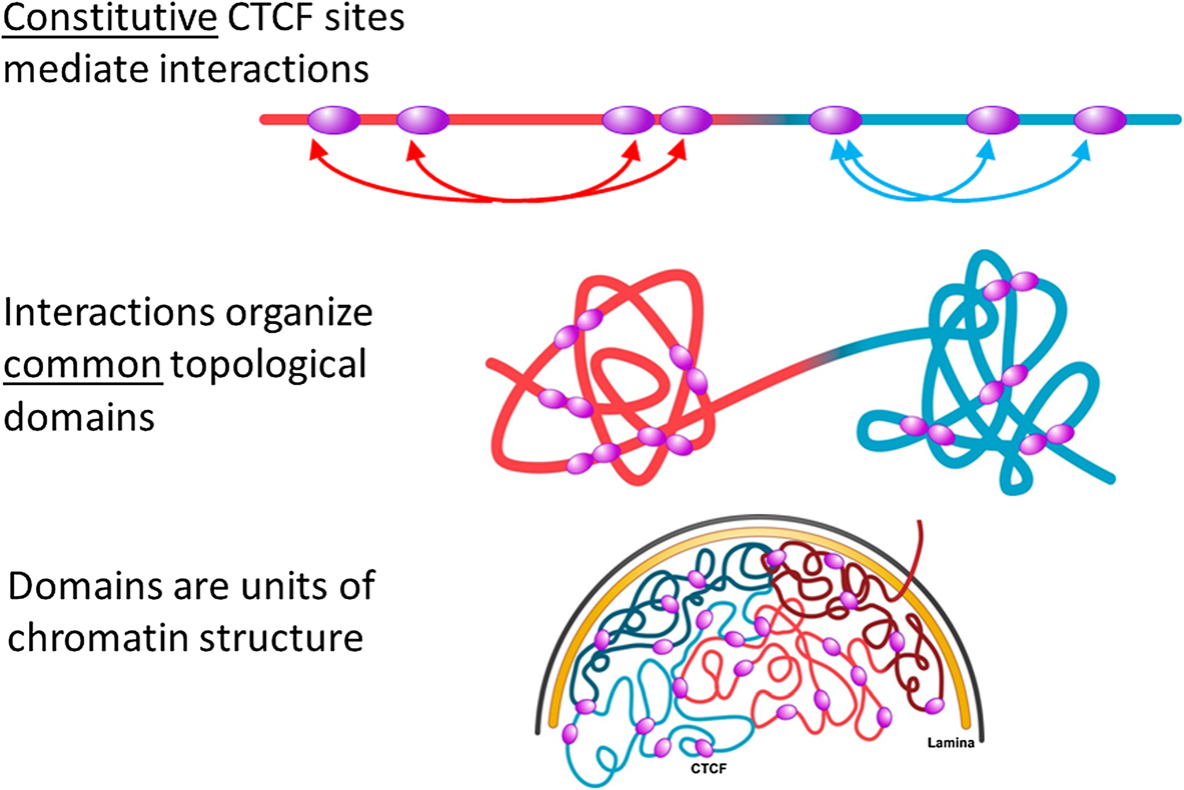
**A proposed model of role of cCTCF loci in chromatin structure.** CTCF, cohesin (not shown) and possibly other factors such as Znf143 and mediator [57] (not shown) mediate long-range chromatin interactions through the constitutive CTCF sites. The cCTCF-mediated interactions organize/maintain the topological domains [23] that are units of chromosome organization. In this conceptual diagram, only constitutive CTCF sites are shown (purple ovals) and the topological domains are coloured individually.

Our analysis in cancer cell lines K562 and MCF7 further revealed that the majority of the cCTCF sites were located in the CTCF-mediated chromatin interactions from ChIA-PET [24]. The proportion of the cCTCF sites in the chromatin interactions was higher for those cCTCF sites that overlapped with cCohesin loci than for those that did not. These results suggest that the genomic loci that are *constitutively* co-bound by both CTCF and cohesin may be involved in establishing or maintaining the “common” or “ground state” chromatin architecture in most human cell lines (Figure 8). This idea is consistent with the finding that the overall topological domain structure between cell/tissue types or across species is largely unchanged [23]. Hu et al. further suggested that the geometric shapes of the topological domains are strongly correlated with several genomic and epigenetic features [55]. We found that most CTCF-mediated interactions from ChIA-PET [24] involved cCTCF and were within a domain. It is conceivable that the cCTCF/cCohesin sites are an integral part of the large, discrete domains [23,24], possibly mediating/maintaining the sub-domain structures within a domain.

## Conclusions

Using ENCODE ChIP-seq data we identified ~450,000 CTCF binding sites in CTCF peaks from 56 cell lines. We also identified ~24,000 cCTCF and ~12,000 cCTCF/cCohesin binding sites. The cCTCF sites were located in unique genomic environments and were over-represented in CTCF-mediated global chromatin interactions that are predominantly within, but not between, the proposed topological domains. We suggest that CTCF and cohesin cooperate in those loci to establish/maintain the “common” chromatin structure in most human cell lines.

## Methods

### Datasets

We downloaded all ChIP-seq data defined as either narrow or broad peaks from the ENCODE portal at the UCSC genome browser (http://genome.ucsc.edu/ENCODE/downloads.html). We extended/trimmed all TFBS ChIP-seq peaks to 200 bp in length from the center of the peak. All genomic sequence data, such as CpG islands, were downloaded from the UCSC genome browser. All data were in GRCh37 (hg19) assembly.

### Predicting CTCF binding sites in ChIP-seq data

For each ChIP-seq dataset, we predicted the location of a CTCF binding site in each peak using the GADEM software [56]. The position weight matrix (PWM) model for CTCF was obtained from earlier *de novo* analysis [56] of the CTCF ChIP-seq datasets [31]. The *p*-value for the PWM score cutoff was set to 0.0005 to identify well-defined CTCF sites. For each ChIP-seq peak, we selected the single highest scoring site that passed the *p*-value cutoff for the PWM score as the binding site. We found a CTCF binding site in 80-95% of the CTCF ChIP-seq peaks in each dataset. We combined binding sites from replicate experiments for any cell line and retained only the unique ones. Similarly, we predicted the CTCF binding sites in all other ChIP-seq datasets from ENCODE. The coordinates of all unique CTCF binding sites identified in the 112 CTCF ChIP-seq datasets in 56 cell lines are provided in Additional file 5.

### Motif conservation analysis

We extracted the 46-way multiZ alignments (hg19) for the 23,709 cCTCF binding sites (16 bp) plus the 10 bp flanking regions using the Galaxy “Extract MAF blocks given a set of genomic intervals” tool (http://main.g2.bx.psu.edu/). Multiple blocks in the Galaxy output were merged using a custom python code. For each multiZ alignment, we scanned each sequence in the alignment for a CTCF binding site using the same PWM and *p*-value (0.0005) cutoff as before. We then counted the number of sequences (equivalently, species) in each alignment containing a CTCF binding site and used the number as a surrogate for conservation. Similarly, we randomly selected 23,709 non-constitutive CTCF binding sites that were identified in 2–10 cell lines and repeated the above analysis.

### Overlap with ChIP-PET interaction data

We downloaded genome-wide CTCF-mediated chromatin interactions identified by ChIA-PET in K562 and MCF7 cell lines from ENCODE/GIS-Ruan (http://hgdownload.cse.ucsc.edu/goldenPath/hg19/encodeDCC/wgEncodeGisChiaPet/). The CTCF binding sites used were those we predicted from ENCODE CTCF ChIP-seq peaks in K562 and MCF7 as described above. There were 25,304 unique CTCF-mediated interactions from cell line K562. For cell line MCF7, two replicate experiments yielded 50,498 and 20,140 unique CTCF-mediated interactions, respectively. Each interaction is defined by a pair of genomic coordinates, referred to herein as region1 and region2, respectively. Since is an interaction has no directionality, the order of the two regions is irrelevant. It is worth pointing out that a region in one interaction pair may overlap with region(s) in another.

### Proportions of CTCF sites in ChIA-PET detected interactions

We found 23,577 cCTCF and 81,464 CTCF excluding cCTCF (non-constitutive CTCF) binding sites in cell line K562 based on the ChIP-seq data. We then counted the number of cCTCF and non-constitutive CTCF sites contained within any regions in the ChIA-PET interaction data.

### Proportion of ChIA-PET detected interactions involving CTCF sites

A ChIA-PET interaction region may contain a cCTCF site, a non-constitutive CTCF site, or neither. Thus, a ChIA-PET interaction pair may be one of six possible types: cCTCF and cCTCF, cCTCF and non-constitutive CTCF, cCTCF and neither, non-constitutive CTCF and non-constitutive CTCF, non-constitutive CTCF and neither, neither and neither (Table 4). Since a region in the interaction pair may contain multiple cCTCF and/or non-constitutive CTCF sites, we assigned all possible types of interactions possible for the pair of regions and counted them proportionally. For example, if region 1 contained one cCTCF and one non-constitutive CTCF site and region 2 contained one non-constitutive CTCF site, we assigned one-half count for cCTCF and non-constitutive CTCF interaction and one-half count for non-constitutive CTCF and non-constitutive cCTCF interaction. This way, each interaction pair in the original ChIA-PET data contributes equally and the sum of the counts equals to the total number of interaction pairs in the original ChIA-PET data.

## Abbreviations

CTCF: CCCTC binding factor; Rad21: RAD21 homolog (*S*. *pombe*); Smc1: Structure maintenance of chromosomes 1; Smc3: Structure maintenance of chromosomes 3; cCTCF: Constitutive CTCF sites; cRad21: Constitutive Rad21 site; cSmc3: Constitutive Smc3 site; cCTCF/cCohesin: Constitutive CTCF sites that overlap with constitutive cohesin loci; cCTCF-non-cohesin: Constitutive CTCF sites that did not overlap with either Rad21 or Smc3 loci.

## Competing interests

The authors declare that they have no competing interests.

## Authors’ contributions

LL conceived the study, YL, WH, LN and LL performed the analyses, and DMU and SC were involved in design, analysis and interpretation of data. All authors contributed and approved the final manuscript for publication.

## Supplementary Material

Additional file 1 Supplementary Text. Click here for file

Additional file 2: Table S1 Summary results for CTCF, Rad21, Smc3 and Znf143. **Table S2.** Comparison of the proportion of overlap (fraction of bins containing peaks for both proteins among bins containing peaks for at least one of the proteins) among constitutive sites versus non-constitutive sites. **Table S3.** Association between CTCF and Rad21/Smc3 and Rad21/Znf143 and Smc3/Znf143. Click here for file

Additional file 3: Figure S1 The Venn diagrams showing the pair-wise overlap between CTCF and Rad21, Smc3, and Znf143 when both are constitutive or non-constitutive. Counts provide for each region in the Venn diagrams. **Figure S2**. The Venn diagrams showing the trio-wise overlap between CTCF and Rad21 and Smc3, Rad21 and Znf143, and Znf143 and Smc3 when both are constitutive or non-constitutive. **Figure S3**. Box plots of the fraction of overlap within each cell line between classes of CTCF sites and various factors/features. **Figure S4.** Density of epigenetic marks cCTCF/cCohesin loci (top panels, black) and the cCTCF-Cohesin loci (bottom panels, red) in Gm12878, Helas3, Hepg2, and K562 cell lines. Click here for file

Additional file 4 Coordinates of constitutive CTCF binding sites. Click here for file

Additional file 5 **Coordinates of all unique CTCF binding sites identified in the 112 CTCF ChIP-seq datasets in 56 cell lines.** The first three columns list the coordinates whereas the third column lists the strand in which the site was found. The last column lists the number of cell lines in which the site was identified. All coordinates are in GRCh37 build. Click here for file
