# Supplementary material for: Characterization of constitutive CTCF/cohesin loci: a possible role in establishing topological domains in mammalian genomes
Source: BMC Genomics. 2013 Aug 14;14:553. doi: 10.1186/1471-2164-14-553 (PMC3765723; doi:10.1186/1471-2164-14-553)
Supplement: Additional file 1 — Supplementary Text. [file 1471-2164-14-553-S1.docx]

Supplementary material for

**Characterization of constitutive CTCF/Cohesin loci: a possible role in establishing topological domains in mammalian genomes**

Yuanyuan Li^1^, Weichun Huang^1^, Liang Niu^1^, David M. Umbach^1^ Shay Covo^2^ and Leping Li^1,*^

^1^Biostatistics Branch, ^2^Laboratory of Molecular Genetics, National Institute of Environmental Health Sciences, Research Triangle Park, NN 27709, USA

1. **The association between CTCF and Rad21, Smc3 or Znf143 is strongest when all are constitutive**

The ENCODE consortium provides 114 ChIP-seq datasets for CTCF in 56 cell lines, 15 for Rad21 in 6 cell lines, 4 for Smc3 in 4 cell lines, and 4 for Znf143 in 4 cell lines. We conceptualized each chromosome as segmented into bins of equal size. For each data set, we counted a protein as present in a bin if the center of its ChIP-seq peak occurred within that bin (operationally, we divided the location of the center by the bin width, the integer part then gave the bin number). If we had multiple data sets for a single protein from a given cell line, we created a summary for that protein in that cell line by counting a bin as containing a peak if it contained a peak in at least one of the replicate datasets. Then, for each protein, we summed the counts in each bin across cell lines. To avoid potential false positives, for each protein separately, we declared bins with fewer than two peak centers (0 or 1) as empty for that protein. Although we carried out analyses with four different bin sizes (100, 200, 300, and 400bp), we only show the results for the bin size of 200bp as the results for all four bin sizes are consistent.

Analogous to the definition in the main text, we referred to a bin that contains peak centers from more than 90% of the cell lines for a protein as a “constitutive” bin for that protein. All the remaining bins were referred to as “non-constitutive”. Using this binning approach, we counted the number of bins containing each protein (CTCF and Rad21, Smc3, or Znf143) dichotomized by the constitutive versus non-constitutive designation. The total number of bins in which any protein was present was largest for CTCF and smaller for the other proteins, for which many few data sets were available (**Table S1)**.

Using this binning approach, we also counted the number of bins containing both constitutive CTCF and Rad21, Smc3, or Znf143 and conversely, the number of bins containing both non-constitutive CTCF and Rad21, Smc3, or Znf143. The pairwise overlap between CTCF and each of the other three proteins (that is, the number of bins containing ChIP-seq peaks for both proteins as a fraction of bins containing ChIP-seq peaks for at least one of them) was larger when both proteins were constitutive than when both were non-constitutive (**Figure S1** and **Table S2)**. A similar relationship was evident when we considered the overlap of CTCF with two of the other proteins simultaneously (**Figure** **S2** and **Table S3)**.

In summary, we showed that the association between CTCF and cohesin (Rad21/Smc3) and between CTCF and Rad21/Smc3/Znf143 was strongest when all are constitutive.

1. **cCTCF/cCohesin loci have distinct histone modification profiles**

To compare the epigenetic environments between the cCTCF/cCohesin and the cCTCF-Cohesin sites, we computed the density of 12 histone marks (Supplemental Figure S4) in those loci in the four cell lines (Gm12878, Helas3, Hepg2, and K562 cell lines) for which both Rad21 and Smc3 ChIP-seq data were available. CTCF sites are surrounded by 10 well-positioned nucleosomes at each side in individual cell line [[1-3](#_ENREF_1)]. Here we showed that this relationship is also true for both the cCTCF/cCohesin and the cCTCF-Cohesin loci. Those two profiles almost completely overlapped with each other and match the previous published results [[1-3](#_ENREF_1)].

The nucleosomes with the H3k4me1 mark in the cCTCF/cCohesin loci were well-positioned—in a startling contrast to the fuzzy H3k4me1 nucleosome in the cCTCF-Cohesin loci (**Figure** **S4**). Conversely, the cCTCF/cCohesin loci had low H3k4me3 mark whereas the same mark for the cCTCF-Cohesin loci was high. Moreover, the cCTCF-Cohesin loci were also enriched with other transcriptionally active marks such as H3k4me2, H3k9ac, H3k27ac, and H3k79me2 compared to the cCTCF/cCohesin loci.

1. **cCTCF/cCohesin loci are associated with cZnf143 loci**

We found that about 37% of 12,014 cCTCF/cCohesin sites overlapped with constitutive Znf143 peaks in four cell lines. Moreover, 56% of 8,038 constitutive CTCF sites in Znf143 peaks (cZnf143 sites) overlapped with cCTCF/cCohesin sites. In comparison, only 2.9% (27) of them overlapped with the 925 cCTCF-cohesin sites. This result indicates that the cZnf143 loci were associated with the cCTCF/cCohesin loci (*p*-value<10^-16^, binomial test).

A genome-wide Znf143 occupancy study revealed that Znf143 binding sites are enriched in bidirectional promoters (a pair of promoters arranged in a head-to-head orientation) and are largely located near the TSS [[4](#_ENREF_4)]. In contrast, ~85% of the cZnf143 sites in our analysis are greater than 5kb from the TSS and lacked CpG islands (data not shown).

We refer to the 4,481 cZnf143 sites that overlapped with the cCTCF/cCohesin sites as the cCTCF/cCohesin/cZnf143 sites. Gene ontology (GO) analysis showed that the genes associated with the cCTCF/cCohesin/cZnf143 loci were significantly enriched with GO terms such as cell adhesion (multiple testing adjusted *p*-value=1.7×10^-7^), anatomical structure development (*p*-value=1.3×10^-5^), multicellular organismal development (*p*-value=3.7×10^-5^), and cellular developmental process (*p*-value=4.2×10^-4^).

**Supplementary methods**

**Gene Ontology**

Gene ontology was carried out on DAVID (<http://david.abcc.ncifcrf.gov/>) [[5](#_ENREF_5)]. A CTCF site was assigned to a gene if it was located between the transcription start and end sites or within 5k upstream from the transcription start site of the gene using the UCSC refGenes model. The unique gene symbols for the set of CTCF sites were subjected to DAVID for gene ontology analysis.

**Epigenetic profile analysis of CTCF flanking regions**

For each subset of CTCF binding sites, e.g., cCTCF/cCohesin or cCTCF-cohesin, we generated an enrichment profile for each of the 11 histone modifications: H3k27ac, H3k9ac, H3k79me2, H3k27me3, H3k9me3, H3k36me3, H4k20me1, H3k4me1, H3k4me2, and H3k4me3. In addition, we analysed the location profiles for Ezh2, p300, H2A.z, and nucleosome. All ChIP-seq BAM data files were downloaded from the ENCODE portal at the UCSC genome browser <http://hgdownload.cse.ucsc.edu/goldenPath/hg19/encodeDCC/>. Specifically, the histone modification, nucleosome, and p300 ChIP-seq data were from wgEncodeBroadHistone, wgEncodeSydhNsome, and wgEncodeHaibTfbs and wgEncodeSydhTfbs, respectively. For each analysis, we calculated the total depth of read coverage surrounding each CTCF site (-2kb to +2kb). We normalized the total depth of read coverage by the number of CTCF sites in the group to obtain the average depth of read coverage per CTCF site.

**References**

1. Cuddapah S, Jothi R, Schones DE, Roh TY, Cui K, Zhao K: **Global analysis of the insulator binding protein CTCF in chromatin barrier regions reveals demarcation of active and repressive domains**. *Genome research* 2009, **19**(1):24-32.

2. Fu Y, Sinha M, Peterson CL, Weng Z: **The insulator binding protein CTCF positions 20 nucleosomes around its binding sites across the human genome**. *PLoS genetics* 2008, **4**(7):e1000138.

3. Rach EA, Winter DR, Benjamin AM, Corcoran DL, Ni T, Zhu J, Ohler U: **Transcription initiation patterns indicate divergent strategies for gene regulation at the chromatin level**. *PLoS genetics* 2011, **7**(1):e1001274.

4. Anno YN, Myslinski E, Ngondo-Mbongo RP, Krol A, Poch O, Lecompte O, Carbon P: **Genome-wide evidence for an essential role of the human Staf/ZNF143 transcription factor in bidirectional transcription**. *Nucleic acids research* 2011, **39**(8):3116-3127.

5. Huang da W, Sherman BT, Lempicki RA: **Systematic and integrative analysis of large gene lists using DAVID bioinformatics resources**. *Nature protocols* 2009, **4**(1):44-57.
