# Supplementary material for: Characterization of constitutive CTCF/cohesin loci: a possible role in establishing topological domains in mammalian genomes
Source: BMC Genomics. 2013 Aug 14;14:553. doi: 10.1186/1471-2164-14-553 (PMC3765723; doi:10.1186/1471-2164-14-553)
Supplement: Additional file 2: Table S1 — Summary results for CTCF, Rad21, Smc3 and Znf143. Table S2. Comparison of the proportion of overlap (fraction of bins containing peaks for both proteins among bins containing peaks for at least one of the proteins) among constitutive sites versus non-constitutive sites. Table S3. Association between CTCF and Rad21/Smc3 and Rad21/Znf143 and Smc3/Znf143. [file 1471-2164-14-553-S2.docx]

Note that the results in the following Tables were all based on a binning method with a bin size of 200bp (see supplementary text for details). The *p*-values were based on binomial proportional test.

**Table S1**. Summary results for CTCF, Rad21, Smc3 and Znf143.

| Protein | Number of bins | | | Proportion | |
| --- | --- | --- | --- | --- | --- |
|  | Constitutive | Non-constitutive | Total | Constitutive | Non-constitutive |
| CTCF | 19,224 | 362,438 | 381,662 | 5.0% | 95.0% |
| Rad21 | 20,148 | 73,167 | 93,315 | 21.6% | 78.4% |
| Smc3 | 14,420 | 33,147 | 47,567 | 30.3% | 69.7% |
| Znf143 | 8,687 | 45,268 | 53,955 | 16.1% | 83.9% |

**Table S2**. Comparison of the proportion of overlap (fraction of bins containing peaks for both proteins among bins containing peaks for at least one of the proteins) among constitutive sites versus non-constitutive sites. Overlap is assessed pair-wise between CTCF peaks and Rad21, Smc3, or Znf143 peaks, respectively

|  | CTCF and Rad21 | | | CTCF and Smc3 | | | CTCF and Znf143 | | |
| --- | --- | --- | --- | --- | --- | --- | --- | --- | --- |
|  | #bins with both | Total | Prop. | #bins with both | Total | Prop. | #bins with both | Total | Prop. |
| Constitutive | 12,741 | 26,631 | 47.8% | 9,976 | 23,668 | 42.1% | 5,076 | 22,835 | 22.2% |
| Non-constitutive | 60,865 | 374,640 | 16.2% | 22,451 | 373,134 | 6.0% | 21,537 | 386,169 | 5.6% |
| P-value | < 2.2×10^-16^ | | | < 2.2×10^-16^ | | | < 2.2×10^-16^ | | |

**Table S3**. Association between CTCF and Rad21/Smc3 and Rad21/Znf143 and Smc3/Znf143

|  | CTCF, Rad21 and Smc3 | | | CTCF, Rad21 and Znf143 | | | CTCF, Smc3 and Znf143 | | |
| --- | --- | --- | --- | --- | --- | --- | --- | --- | --- |
|  | #bins with three | Total | Prop. | #bins with three | Total | Prop. | #bins with three | Total | Prop. |
| Constitutive | 8,795 | 28,086 | 31.3% | 4,205 | 29,588 | 14.2% | 3,556 | 26,758 | 13.3% |
| Non-constitutive | 15,061 | 381,050 | 4.0% | 10,532 | 394,057 | 2.7% | 7,639 | 391,643 | 2.0% |
| P-value | < 2.2×10^-16^ | | | < 2.2×10^-16^ | | | < 2.2×10^-16^ | | |
