# Supplementary material for: Characterization of constitutive CTCF/cohesin loci: a possible role in establishing topological domains in mammalian genomes
Source: BMC Genomics. 2013 Aug 14;14:553. doi: 10.1186/1471-2164-14-553 (PMC3765723; doi:10.1186/1471-2164-14-553)

**Figure S1**. The Venn diagrams showing the pair-wise overlap between CTCF and Rad21, Smc3, and Znf143 when both are constitutive or non-constitutive. Counts provide for each region in the Venn diagrams.

**(a) Constitutive (26,631)**


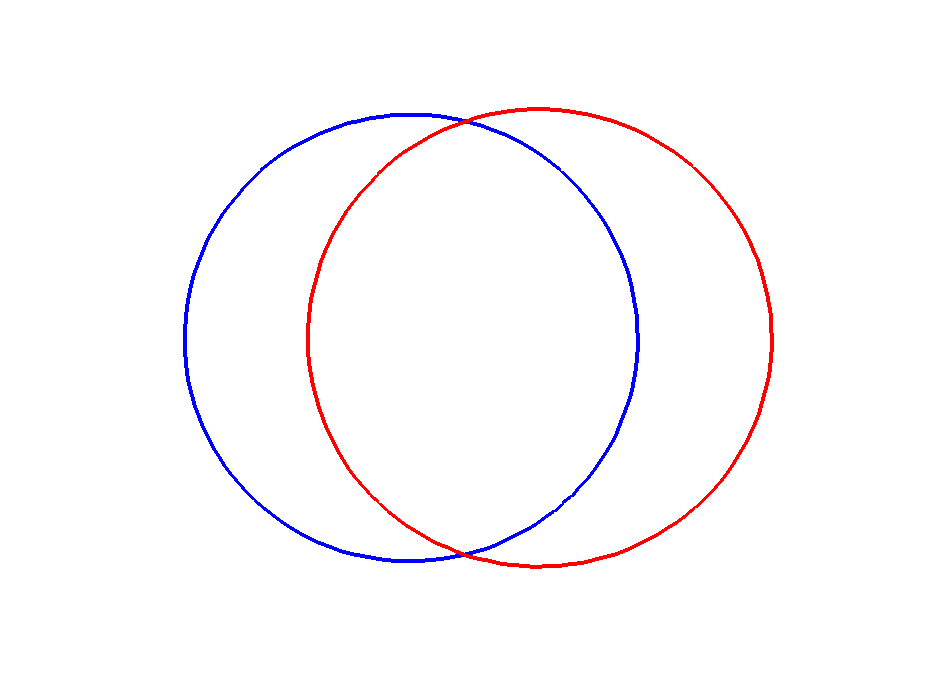


CTCF

19224

7407

12741


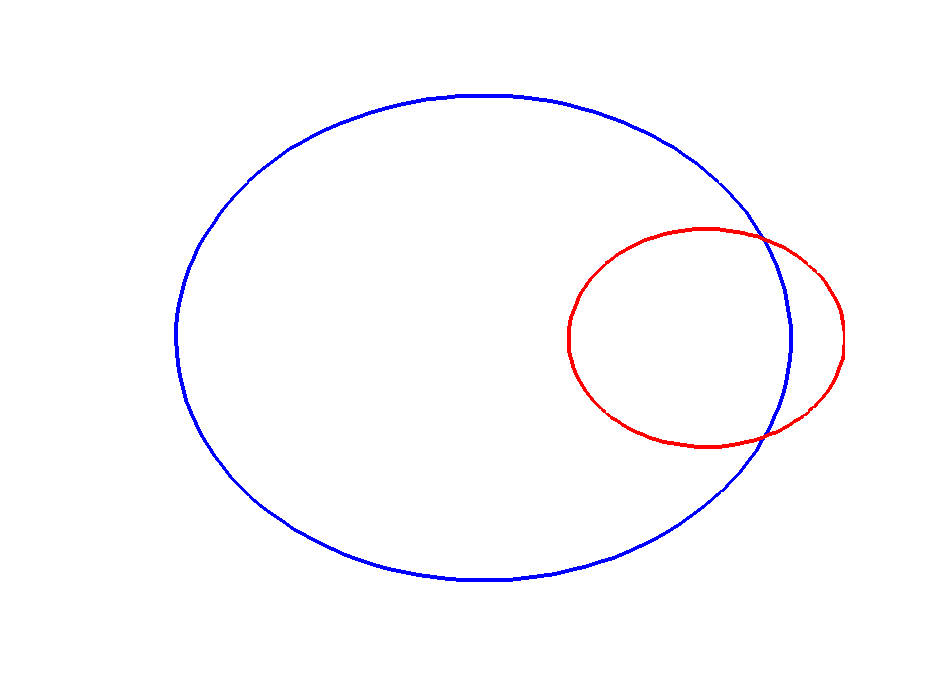


301473

12202

60965

**(a) Non-constitutive (374,640)**

6483

Rad21

20148

CTCF

362438

Rad21

73167


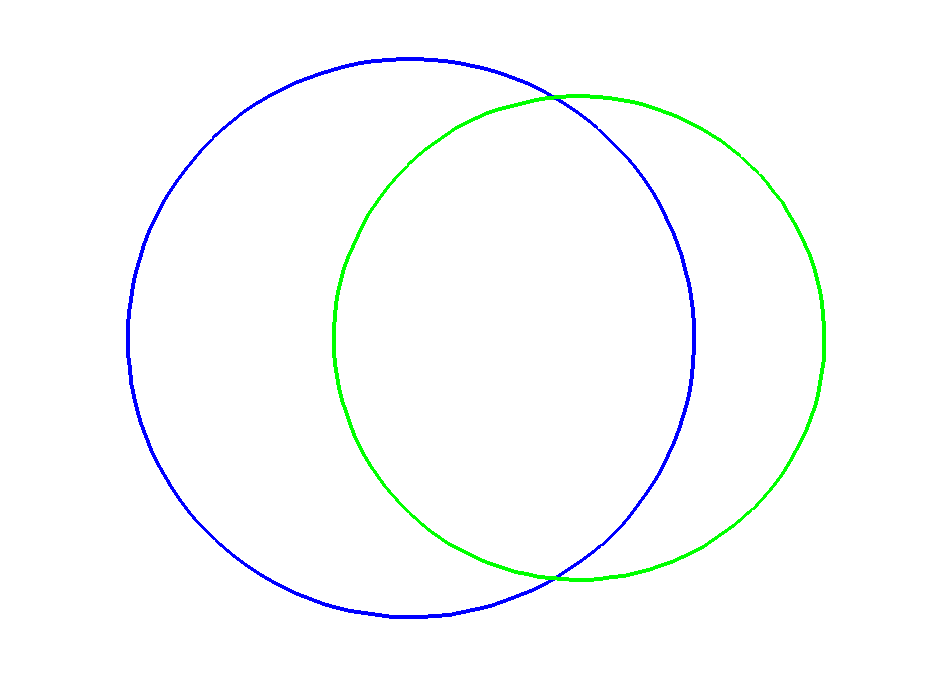


9248

4444

9976


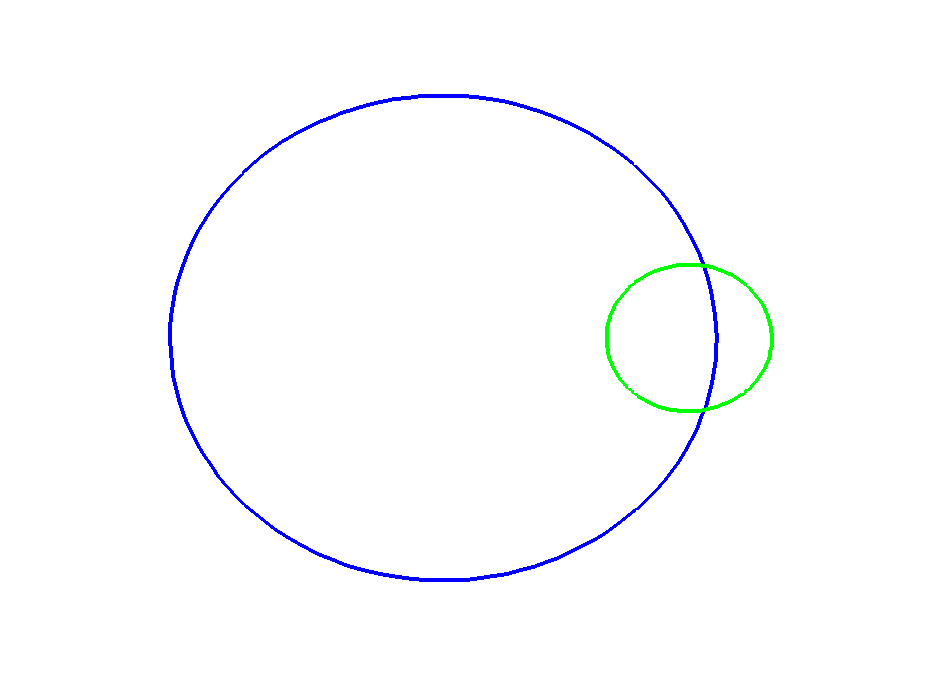


339987

10696

22451

(**b) Constitutive (23,668)**

**(b) Non-constitutive (373,134)**

CTCF

362438

CTCF

19224

Smc3

14420

Smc3

33147


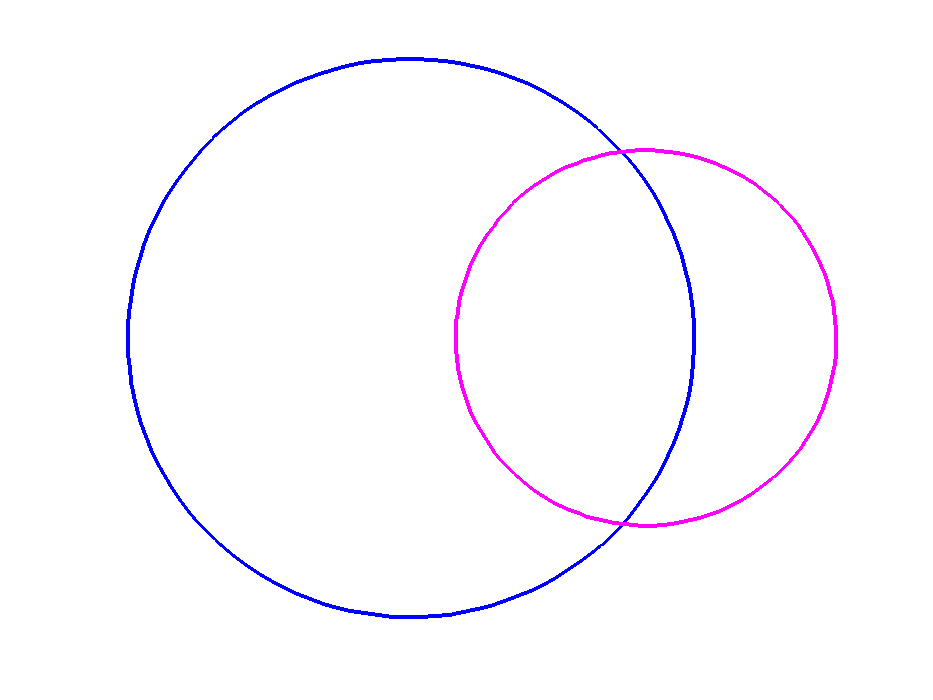

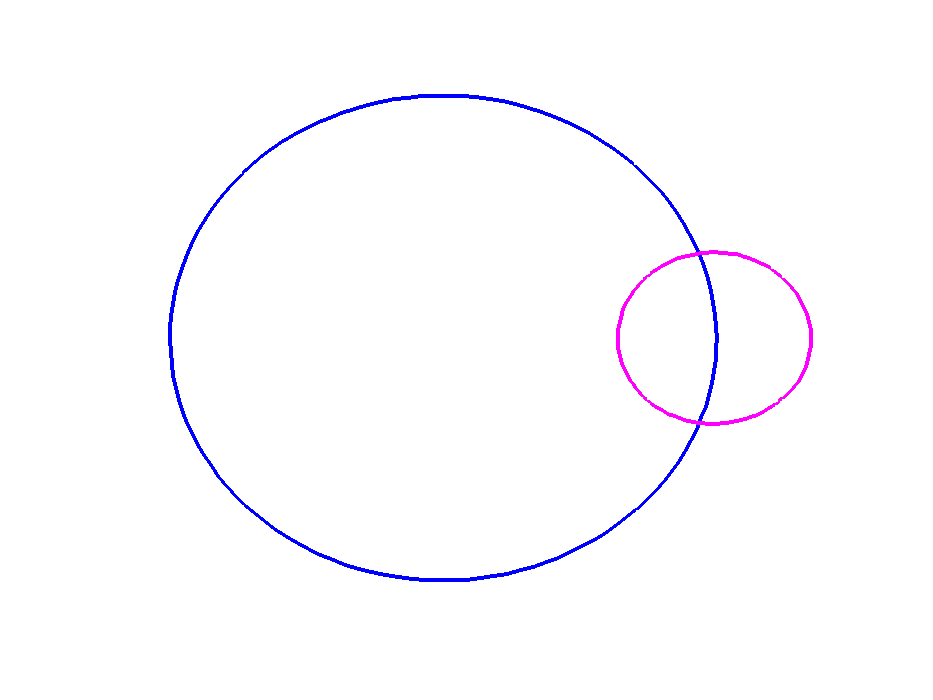


14148

3611

5076

CTCF

362438

Znf143

45268

21537

**(c) Constitutive (22,835)**

**(c) Non-constitutive (386,169)**

Znf143

8687

CTCF

19224

340901

23731

**Figure S2**. The Venn diagrams showing the trio-wise overlap between CTCF and Rad21 and Smc3, Rad21 and Znf143, and Znf143 and Smc3 when both are constitutive or non-constitutive. Counts are provided for regions where all 3 proteins bind (3-way intersection) and where only one protein binds; counts for regions where exactly 2 proteins bind can be calculated.


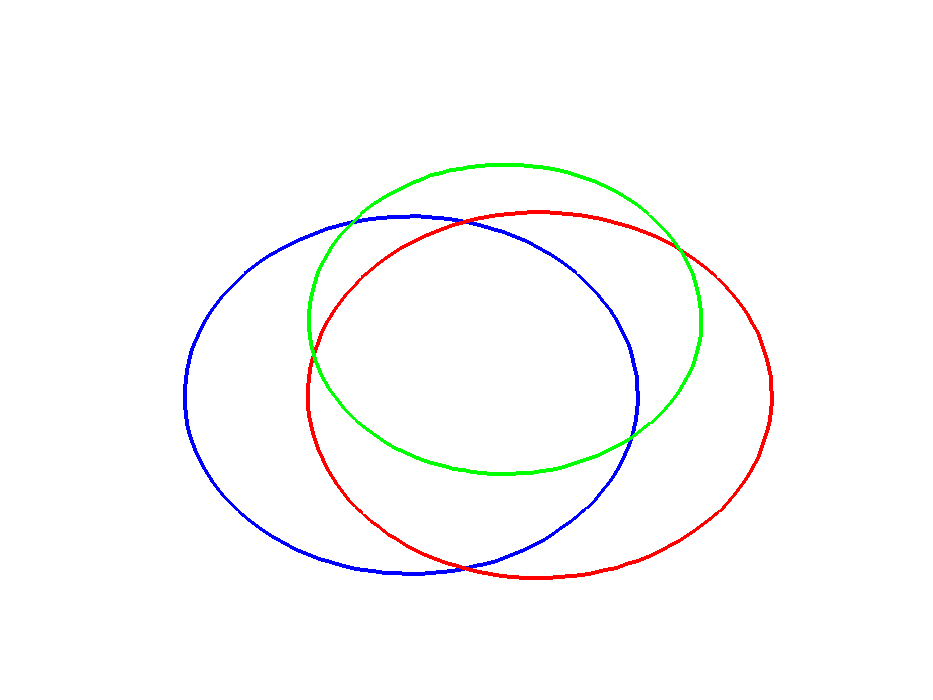

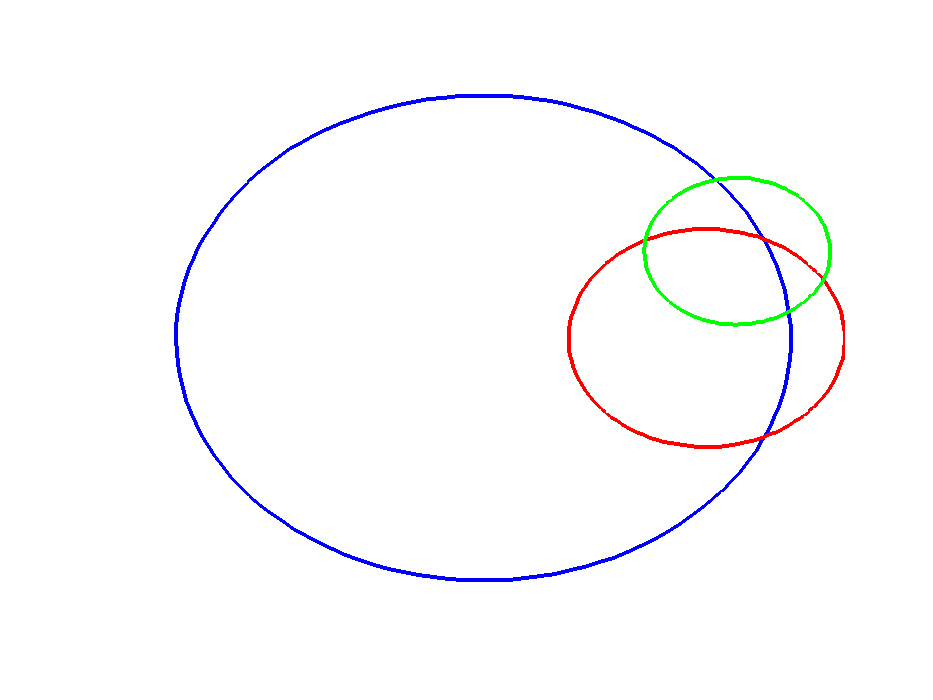


CTCF

19224

Smc3

14420

8795

CTCF

362438

Smc3

33147

15061

**(a) Constitutive (28,086)**

**(a) Non-constitutive (381,050)**

4418

7916

1455

Rad21

20148

5302

294183

Rad21

73167

6310


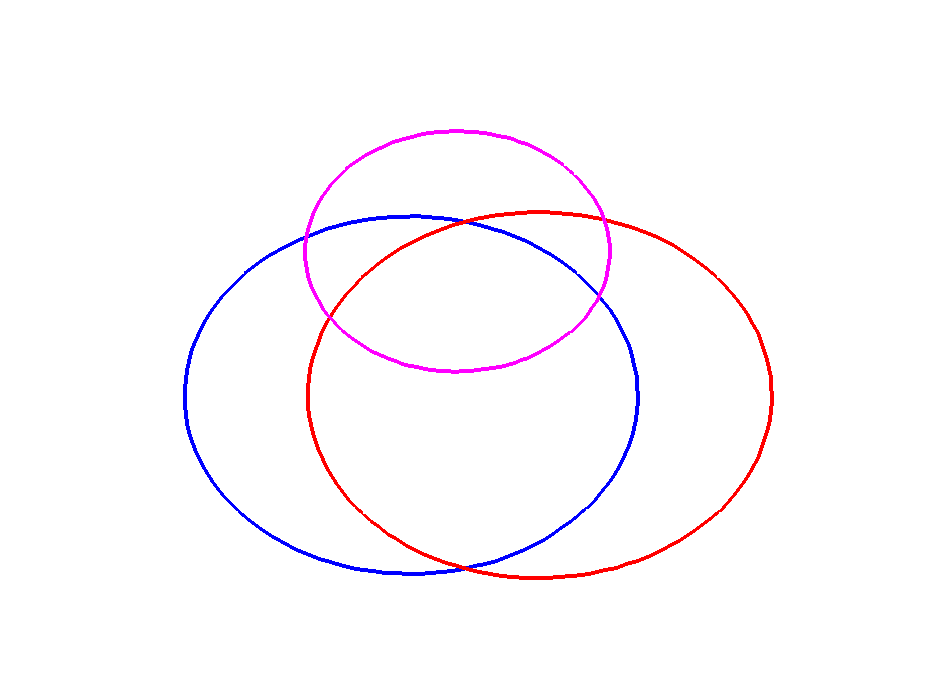


5612

2957

4025

**(b) Constitutive (29,588)**

6753


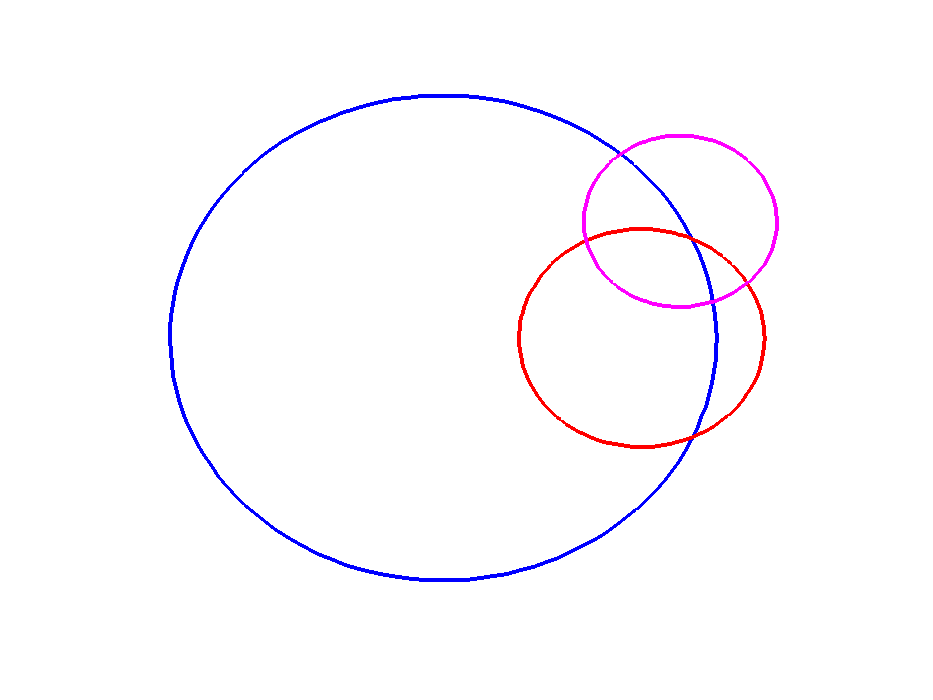


290568

19317

10532

**(b) Non-constitutive (394,057)**

7888

Znf143

8687

CTCF

19224

Rad21

20148

CTCF

362438

Znf143

45268

Rad21

73167


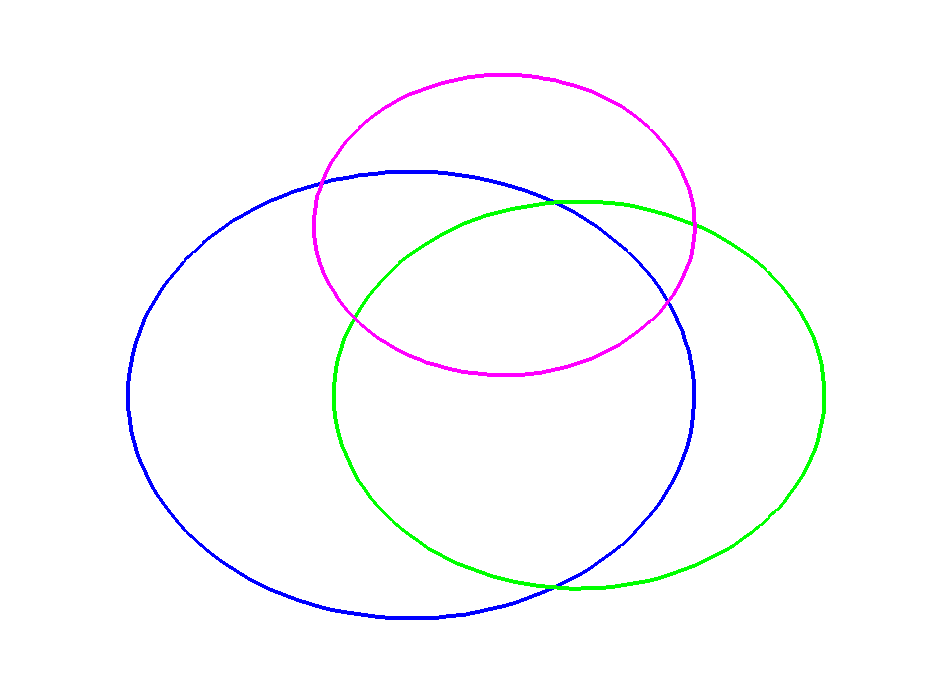


7728

3923

3556

**(c) Constitutive (26,758)**

3090


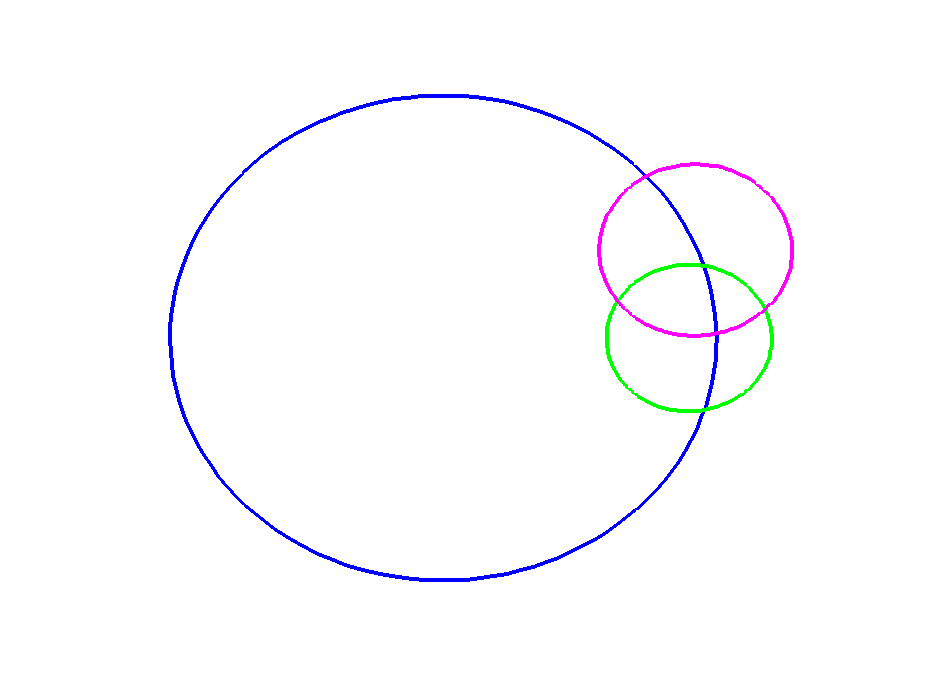


326089

18509

7639

**(c) Non-constitutive (391,643)**

5474

Znf143

8687

Smc3

14420

CTCF

19224

CTCF

362438

Smc3

33147

Znf143

45268

**Figure. S3**. Box plots of the fraction of overlap within each cell line between classes of CTCF sites and various factors/features. The classes of CTCF sites are: cCTCF/cCohesin sites (black), cCTCF-Cohesin sites (red), or all CTCF sites bound in each cell line excluding cCTCF sites (blue). Each point in a box plot represents a cell line.


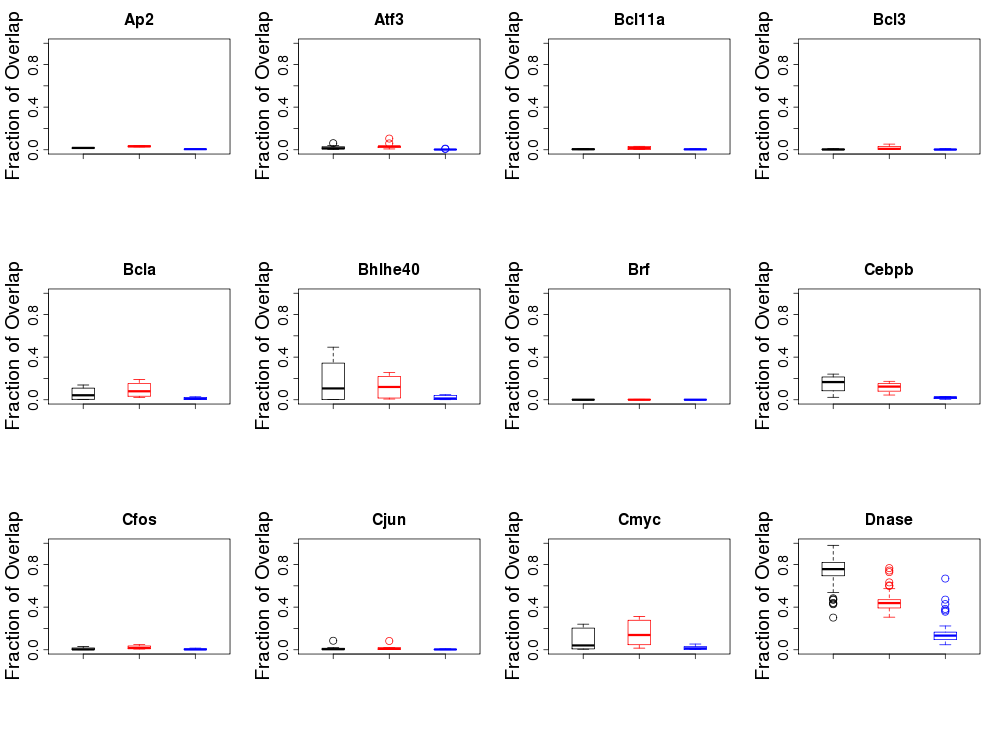


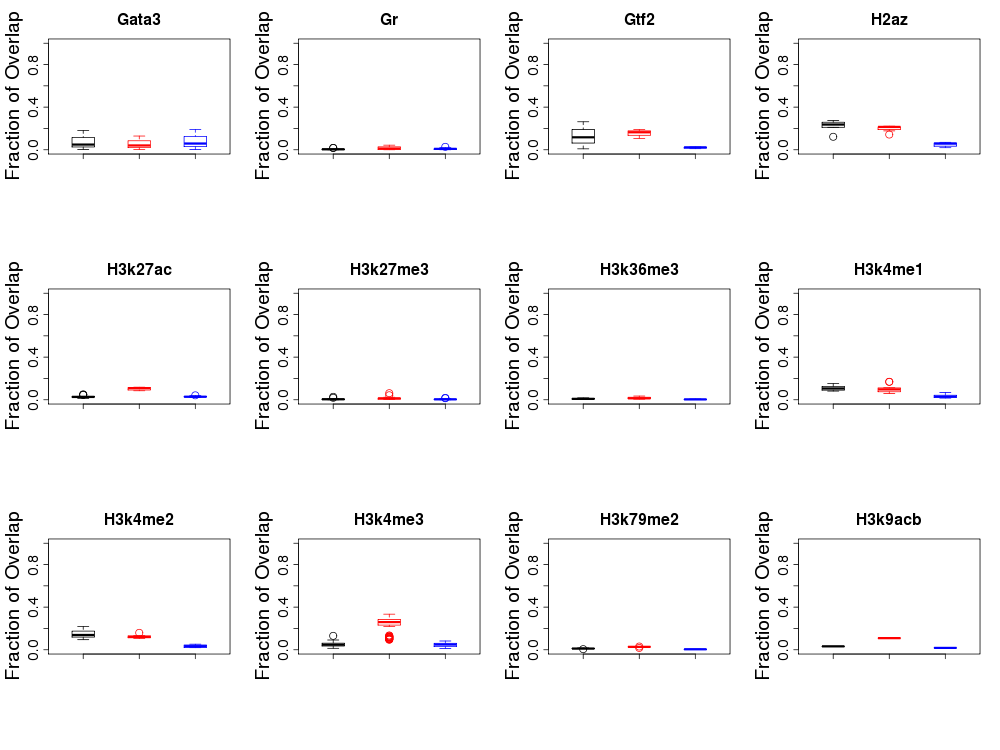


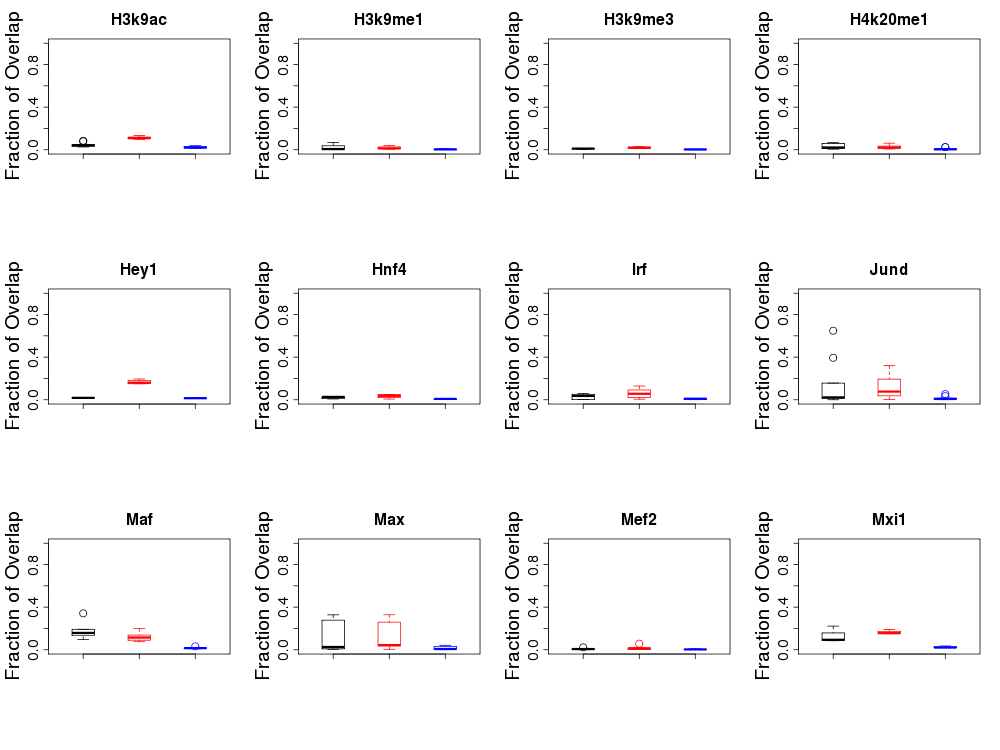


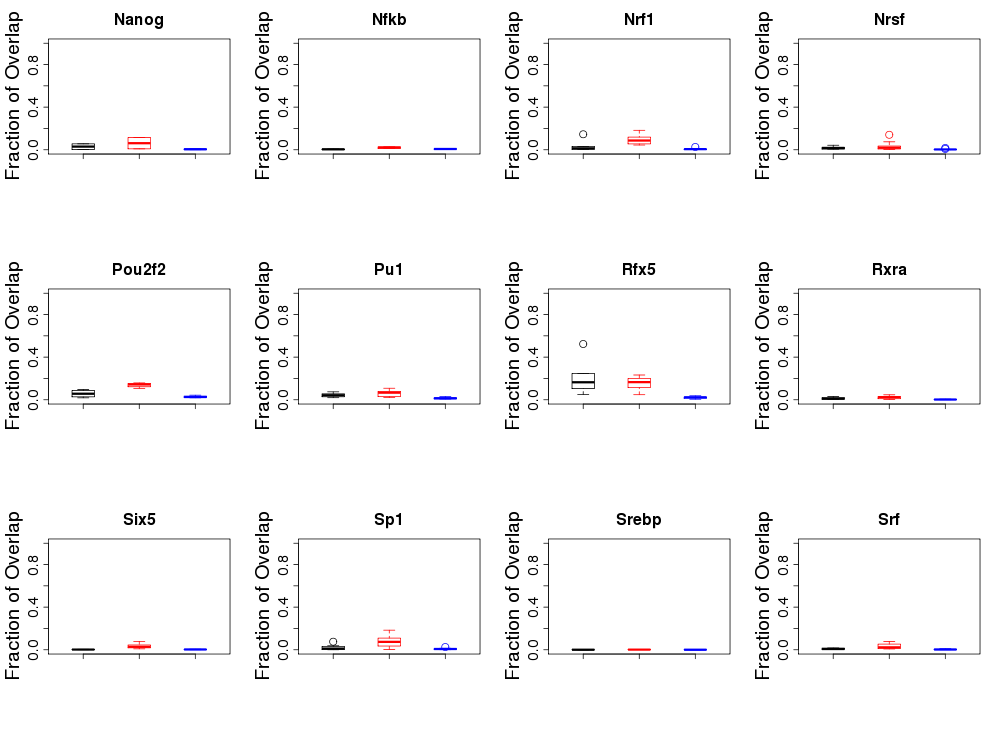


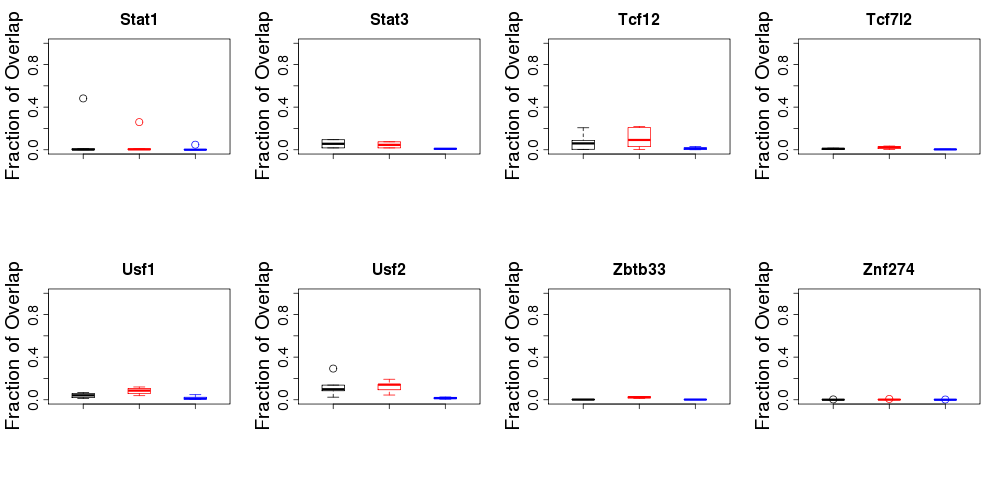


**Figure S4.** Density of epigenetic marks cCTCF/cCohesin loci (top panels, black) and the cCTCF-Cohesin loci (bottom panels, red) in Gm12878, Helas3, Hepg2, and K562 cell lines.


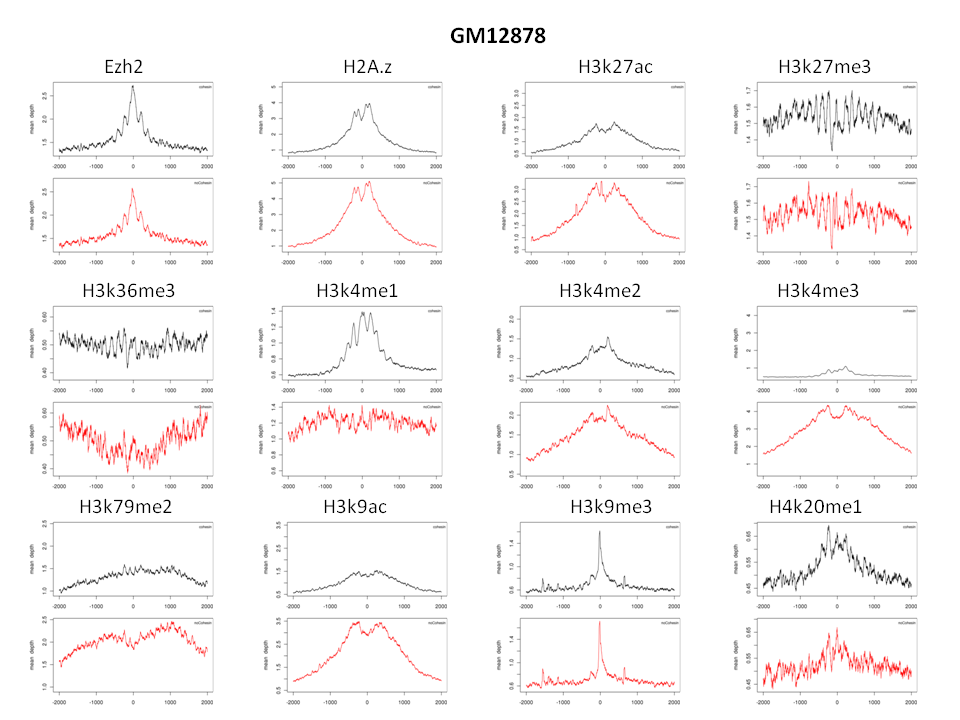


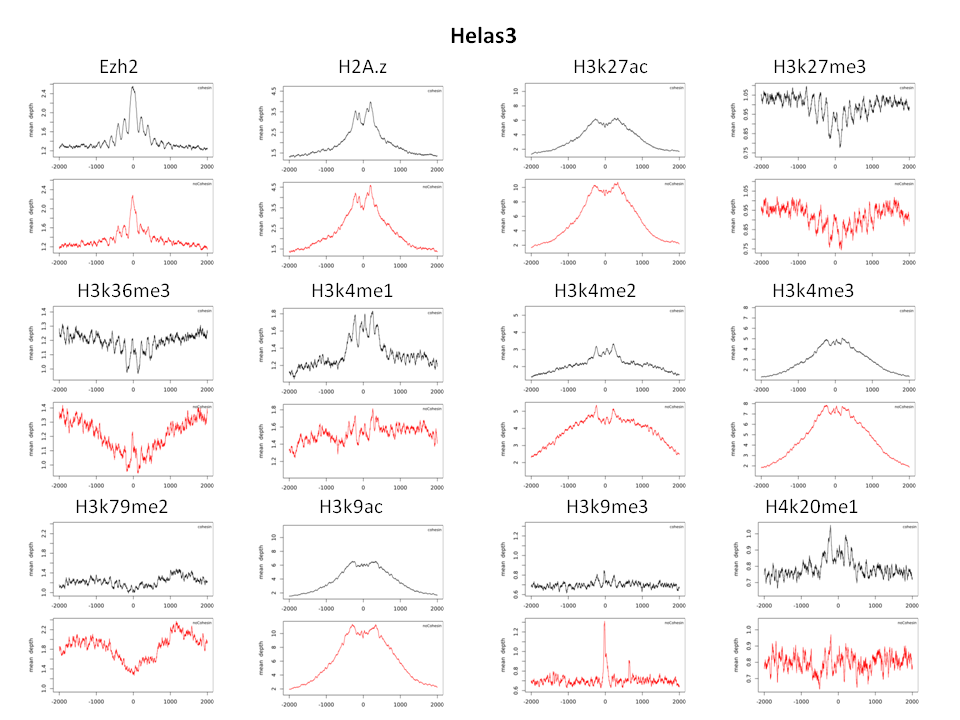


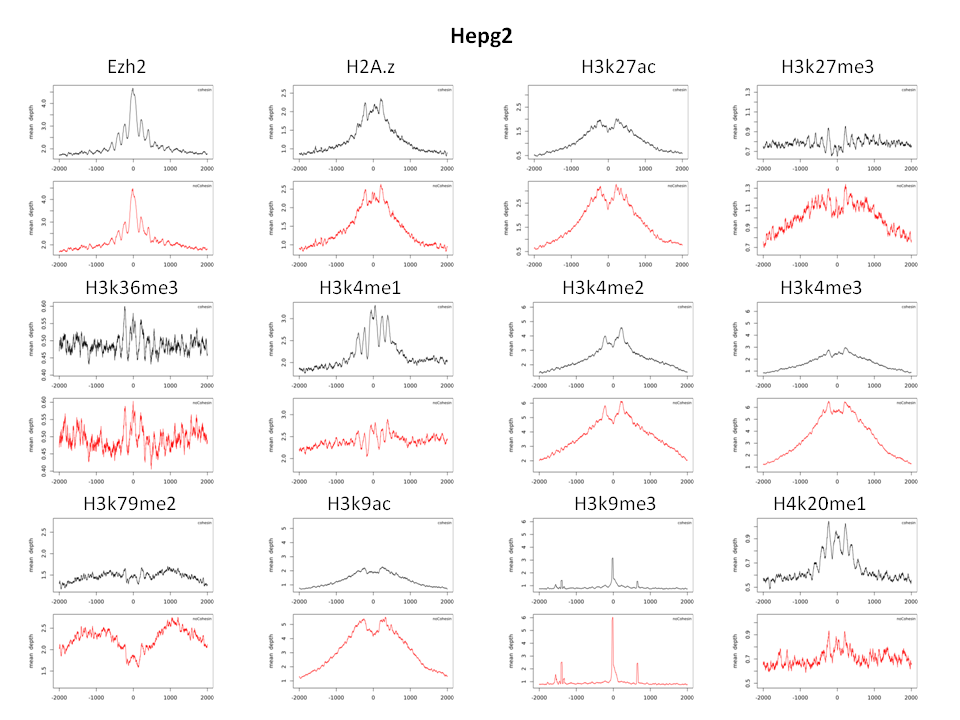


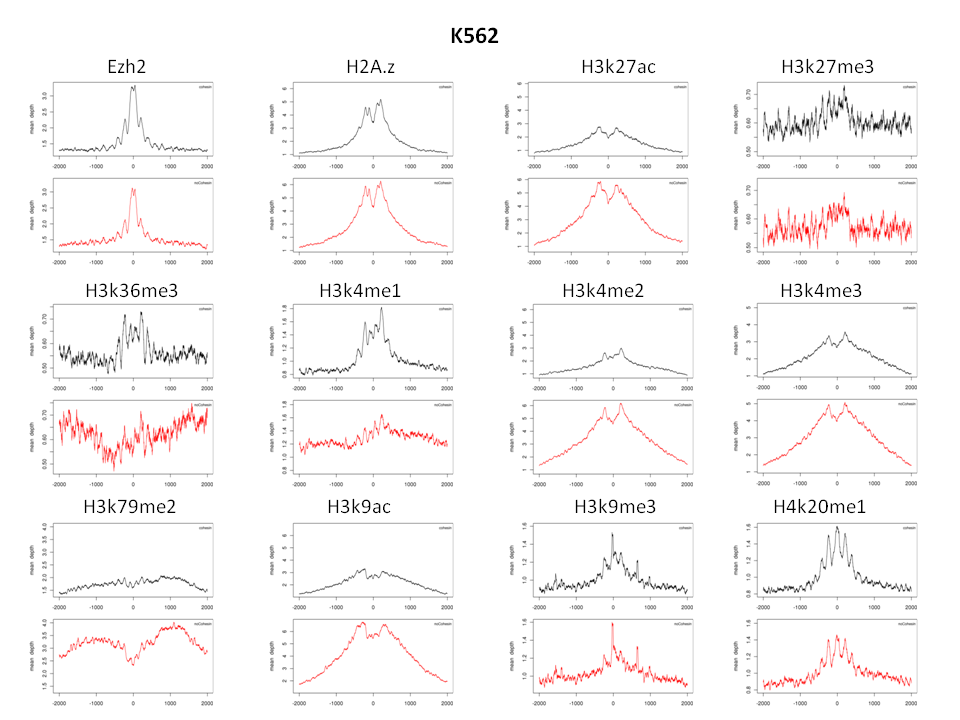

Supplement: Additional file 3: Figure S1 — The Venn diagrams showing the pair-wise overlap between CTCF and Rad21, Smc3, and Znf143 when both are constitutive or non-constitutive. Counts provide for each region in the Venn diagrams. Figure S2. The Venn diagrams showing the trio-wise overlap between CTCF and Rad21 and Smc3, Rad21 and Znf143, and Znf143 and Smc3 when both are constitutive or non-constitutive. Figure S3. Box plots of the fraction of overlap within each cell line between classes of CTCF sites and various factors/features. Figure S4. Density of epigenetic marks cCTCF/cCohesin loci (top panels, black) and the cCTCF-Cohesin loci (bottom panels, red) in Gm12878, Helas3, Hepg2, and K562 cell lines. [file 1471-2164-14-553-S3.docx]
